# Supplementary figures and images for: Could listening to music during pregnancy be protective against postnatal depression and poor wellbeing post birth? Longitudinal associations from a preliminary prospective cohort study
Source: BMJ Open. 2018 Jul 17;8(7):e021251. doi: 10.1136/bmjopen-2017-021251 (PMC6059338; doi:10.1136/bmjopen-2017-021251)

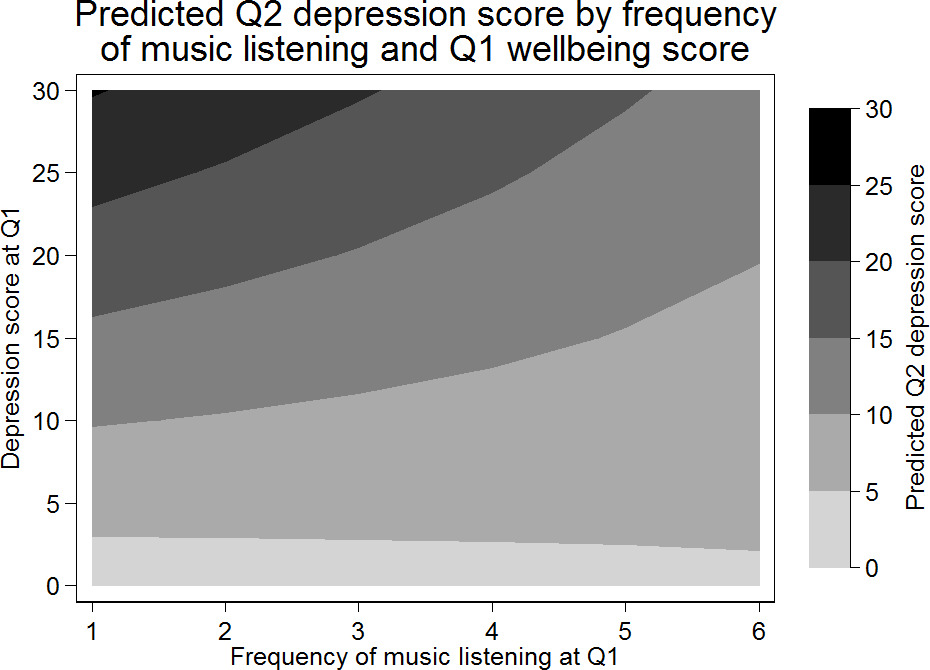

Supplement: Supplementary data [file bmjopen-2017-021251supp001.jpg]

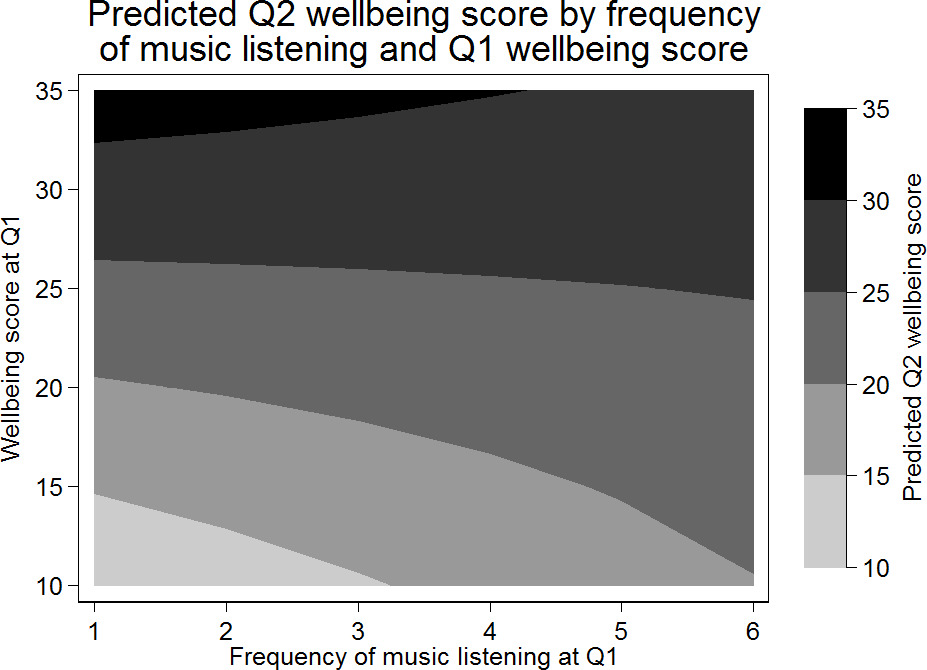

Supplement: Supplementary data [file bmjopen-2017-021251supp002.jpg]
