# Supplementary material for: Could listening to music during pregnancy be protective against postnatal depression and poor wellbeing post birth? Longitudinal associations from a preliminary prospective cohort study
Source: BMJ Open. 2018 Jul 17;8(7):e021251. doi: 10.1136/bmjopen-2017-021251 (PMC6059338; doi:10.1136/bmjopen-2017-021251)
Supplement: Author's manuscript [file bmjopen-2017-021251.draft_revisions.pdf]

BMJ Open

BMJ Open is committed to open peer review. As part of this commitment we make the peer review history of every article we publish publicly available.

When an article is published we post the peer reviewers' comments and the authors' responses online. We also post the versions of the paper that were used during peer review. These are the versions that the peer review comments apply to.

The versions of the paper that follow are the versions that were submitted during the peer review process. They are not the versions of record or the final published versions. They should not be cited or distributed as the published version of this manuscript.

BMJ Open is an open access journal and the full, final, typeset and author-corrected version of record of the manuscript is available on our site with no access controls, subscription charges or pay-per-view fees (http://bmjopen.bmj.com).

If you have any questions on BMJ Open's open peer review process please email
info.bmjopen@bmj.com

BMJ Open

Could listening to music during pregnancy be protective against postnatal depression and poor wellbeing post-birth? Longitudinal associations from a preliminary prospective cohort study.

Journal:	*BMJ Open*
Manuscript ID	bmjopen-2017-021251
Article Type:	Research
Date Submitted by the Author:	19-Dec-2017

Complete List of Authors:	Fancourt, Daisy; University College London Research Department of Epidemiology and Public Health, Department of Behavioural Science and Health<br>Perkins, Rosie; Royal College of Music, Centre for Performance Science; Imperial College London, Faculty of Medicine

Keywords:	MENTAL HEALTH, Depression & mood disorders < PSYCHIATRY, Maternal medicine < OBSTETRICS

SCHOLARONE™
Manuscripts

For peer review only

Could listening to music during pregnancy be protective against postnatal depression and poor wellbeing post-birth? Longitudinal associations from a preliminary prospective cohort study.

Daisy Fancourt PhD [a,b] and Rosie Perkins PhD [b,c]*

[a] Department of Behavioural Science and Health, University College London, London WC1E 7HB

[b] Faculty of Medicine, Imperial College London, London SW7 2AZ

[c] Centre for Performance Science, Royal College of Music, London SW7 2BS

* Corresponding author: Dr Daisy Fancourt, 1-19 Torrington Place, London, WC1E 7HB, d.fancourt@ucl.ac.uk

For peer review only

For peer review only - http://bmjopen.bmj.com/site/about/guidelines.xhtml

Abstract

Objectives: This study explored whether listening to music during pregnancy is longitudinally associated with lower symptoms of postnatal depression and higher wellbeing in mothers post-birth.

Design: Prospective cohort study.

Participants: We analysed data from 395 new mothers aged over 18 who provided data in the third trimester of pregnancy and 3 and 6 months later (0-3 and 4-6 months post birth).

Primary and secondary outcome measures: Listening to music was categorised as 'rarely; a couple of times a week; every day <1hr; every day 1-2hrs; every day 3-5hrs; every day 5+hrs'. Postnatal depression was measured using the Edinburgh Postnatal Depression Scale (EPDS), and wellbeing was measured using the Short Warwick-Edinburgh Mental Wellbeing Scale (SWEMWBS). Multivariate linear regression analyses were carried out to explore the effects of listening to music during pregnancy on depression and wellbeing post birth, adjusted for baseline mental health and potential confounding variables. Sensitivity analyses stratified by baseline mental health and used inverse probability weighting to account for differential non-response.

Results: Listening during pregnancy is associated with higher levels of wellbeing (β=0.40, SE=0.15, 95%CI 0.10 to 0.70) and reduced symptoms of postnatal depression (β=-0.39, SE=0.19, 95%CI -0.76 to -0.03) in the first 3 months post-birth. However, effects disappear by 4-6 months post-birth. These results appear to be particularly found amongst women with lower levels of wellbeing and high levels of depression at baseline and amongst those with a history of mental health conditions.

Conclusions: Listening to music could be recommended as a way of supporting mental health and wellbeing in pregnant women; in particular those who demonstrate low wellbeing or symptoms of PND.

Keywords

Postnatal depression, wellbeing, mental health, music, perinatal

Strengths and limitations of this study

- This preliminary prospective cohort study tracked a sample of women across the perinatal period providing data at 12 week intervals.
- The data include a rich set of variables on music listening behaviours amongst participants.

For peer review only - http://bmjopen.bmj.com/site/about/guidelines.xhtml

- We adjusted for all identified confounding variables in our analyses and ran a series of sensitivity analyses to test our assumptions.
- The data are not nationally representative, although there is a clear spread of participants from varying socio-economic backgrounds as well as variations in the levels of exposure and outcome variables.
- As this is a cohort study and not interventional, it is not possible to confirm csausality.

Funding statement

The study was funded by Arts Council England Research Grants Fund, grant number 29230014 (Lottery). D.F. is supported by the Wellcome Trust [205407/Z/16/Z]. The study was approved by the NHS REC (15/SS/016). The study team acknowledge the support of the National Institute of Health Research Clinical Research Network (NIHR CRN). The authors would like to thank the hospitals involved as Participant Identification Centres as well as Miss Sunita Sharma, Prof Aaron Williamon and Sarah Yorke for their support with the study.

Competing interests statement

Both authors declare no conflict of interest.

For peer review only

Introduction

Perinatal mental health problems affect around 20% of women at some point during the perinatal period (1). In terms of conditions characterised by negative symptomology, postnatal depression (PND) is one of the most common problems, and is a debilitating condition characterised by fatigue, irritability, insomnia and anhedonia; symptoms which in 25% of affected women last for at least one year (2). Over the last two decades, there has been significant research into the effects of PND on mother and infant as well as attention paid to how it can be prevented or managed (3,4). However, in terms of conditions relating more to the absence of positive symptomology, such as low hedonic or eudemonic wellbeing, there has been much less research. The few studies that do exist have found that negative mood, as indicated by the Edinburgh Postnatal Depression Scale, has a correlation of just -0.46 with positive experiences of motherhood (5). This suggests that, as in the wider population, depression and wellbeing are separate constructs in the context of perinatal mental health and a positive perinatal experience is more than simply the inversion of negative mood (6). Building on this, women even without PND have been found to demonstrate impairments in emotional problems and vitality, suggesting that even in the absence of depression mothers can have impaired wellbeing (7). In light of this, a review has argued that psychological well-being defined as a multidimensional construct should be an integral part of maternity care (8), and a more recent construct analysis has highlighted the importance not just of identifying PND but also of identifying women with sub-optimal perinatal wellbeing and supporting them to achieve positive psychological functioning (9).

In seeking to support the perinatal mental health of women, the pregnancy period has been highlighted as critical. Prenatal mental health has repeatedly been highlighted as one of the largest predictors of postnatal depression (10–12) and wellbeing (9). In particular, the third trimester of pregnancy has been identified as an important transition period involving adaptation to emotional and physical changes, leading to feelings of wellbeing often less pronounced than in the previous trimesters (13). Early detection of symptoms of depression and low wellbeing during pregnancy and prompt intervention is therefore important in reducing adverse consequences.

In light of this, there are a number of interventions that have been developed to try and support mental health in the prenatal period as a way of reducing postnatal mental health problems, in particular focusing on the third trimester as a point of intervention. There have been findings that support the application of cognitive-behavioural and interpersonal psychotherapy, suggesting that depression following childbirth could be prevented by brief interventions in the prenatal period (14,15). In exploring other interventions, prenatal hypnotherapy has been found to significantly reduce PND and improve psychological wellbeing at two weeks and ten weeks postpartum (16). And a psychosocial intervention involving group meetings to discuss aspects of parenthood in the final trimester of pregnancy and first 6 months postpartum has been found to reduce PND amongst first-time mothers

For peer review only - http://bmjopen.bmj.com/site/about/guidelines.xhtml

(14). However, as many mothers continue to work full-time until shortly before their due dates, in-person interventions may not be feasible for all mothers and are of course limited by what is available in different geographical areas. As a result, there is a need to identify other home-based interventions that could provide similar mental health support.

Over the past two decades, there has been increasing research showing the effects of listening to music on mental health. A number of reviews have demonstrated the effects of regular music listening including in enhancing mental health in the general population (17), reducing distress in premature infants (18), and reducing stress in adults (19). Specifically in relation to depression, listening to music has been shown to reduce depression amongst adults with chronic pain (20), psychiatric inpatients (21), and older adults (17,22). In relation to wellbeing, music listening has been shown to be associated with better wellbeing not just in controlled interventions but also as a result of ordinary day-to-day listening. A Swedish study involving 500 older adults found correlations between music listening and wellbeing, even when controlling for potential confounding variables (23). Studies tracking daily activities have linked music listening with enhanced wellbeing both in the workplace and in the wider context of people's lives (24,25). Further, music has also been shown to contribute to creating supportive healthy environments, connecting individuals with their emotions and promoting wellbeing (26). Finally, theoretical studies have highlighted the role of music listening in enhancing affect, wellness and resources for recovery and quality of life (27,28). Consequently, both directed music listening interventions and routine day-to-day music listening can affect levels of depression and wellbeing amongst a range of different populations.

Specifically in relation to the perinatal period, a few studies have suggested that music listening may be supportive for mental health. Listening to music for just 30 minutes has been found to reduce cortisol levels and anxiety in pregnant women, leading to recommendations that pregnant women might benefit from regular listening to music as a practice of relaxation (although the effects of regular listening were not tested) (29). A recent study found that women who listened to recorded music for 12 weeks during their pregnancy had significant improvements in anxiety and depression (30). However, the study did not track outcomes postnatally and involved a small sample of women. And a further study has found cross-sectional associations between listening to music and depression and wellbeing amongst new mothers (31). However, this study did not look longitudinally nor involve pregnant women.

Therefore, to date, despite promising results suggesting that listening to music can modulate mental health and wellbeing during the perinatal period, no studies have looked specifically at the impact of listening to music during pregnancy on depression and wellbeing post-birth. In order to address this research gap, this study tracked a cohort of mothers across the perinatal period in order to ascertain

For peer review only - http://bmjopen.bmj.com/site/about/guidelines.xhtml

whether there was a relationship between music listening during pregnancy and postnatal mental health.

Methods

Participants and procedure

This study used data collected as part of a larger study exploring the impact of creative interventions on perinatal mental health. Women living in England in the last trimester of pregnancy (28 weeks or more) and the first nine months post birth (up to 40 weeks) were recruited from hospitals, General Practices, mother and baby charities and through social media in England across October 2015 to March 2016, and completed an anonymous cross-sectional online questionnaire. Women in the final trimester of pregnancy (28 weeks or more) were then invited to continue their participation in a longitudinal study. This involved providing another wave of data 3 months, 6 months and 9 months following the first date of data collection (which equated to providing baseline data T1 during pregnancy, T2 data in the first 3 months post-birth, T3 data in months 4-6 post-birth and T4 data in months 7-9 post-birth). From an initial sample of 550 mothers who consented to be involved in this longitudinal study, a total of 458 mothers provided T2 data (83%), 417 provided T3 data (75.8%) and 392 (71.3%) provided T4 data. The study received ethical approval from the UK NHS Research Ethics Service and all participants gave informed consent prior to involvement in the research.

For this study, we focused on women in the longitudinal study who had provided complete data on the variables we selected for analyses at both T1 and T2: 395 women. We also ran some exploratory follow-up analyses with women who had also provided complete data at T3 (n=307). At T1, women had an average age of 31.9 years (SD=4.9, range 18-47) and an average of 32.9 weeks pregnant (SD=4.1, range 28-42). A total of 69.3% were married, 25.9% were cohabiting with a partner, a further 3.8% were in a relationship but not living together and 1% were single. Of those in relationships, 97.02% reported that their partners worked. Amongst the women, 13.2% had a basic education (equivalent to leaving school at 16), 16.5% had finished education at 18, 41.3% had an undergraduate degree and 29.1% had a postgraduate degree. Just 6.6% reported a household income of less than £16,000, 11.1% reported an income of £16,000-£30,000, 52.9% reported an income of £31,000 to £60,000, 17.5% reported an income of £61,000-90,000 and 11.9% reported an income of over £91,000.

Measures

Symptoms of PND were measured using the Edinburgh Postnatal Depression Scale (EPDS), a 10-item scale used extensively both with pregnant women and new mothers, scored from 0-30 with 10+ indicative of possible symptoms of depression and higher scores of 13+ indicating more severe depression (32).

For peer review only - http://bmjopen.bmj.com/site/about/guidelines.xhtml

Wellbeing was measured using the Short Warwick-Edinburgh Mental Wellbeing Scale (SWEMWBS), a scale that encompasses both hedonic and eudemonic wellbeing comprising 7 items scored from 7 to 35 with higher scores representing higher levels of wellbeing. The raw scores were logit-transformed prior to analysis (33). The New Economics Foundation (NEF) suggests five levels of wellbeing based on quintile analyses of data in the UK Understanding Society Survey, 2009: poor (<22), below average (22-24), average (25-26), good (27-28) and excellent (29-35).

In addition, demographic variables assessed the women's number of weeks pregnant/post-birth, number of other children (0, 1, 2, 3 and 4+), household income (<£16,000, £16,000-£30,000, £31,000-£60,000, £61,000-£90,000, >£90,000), educational attainment (school to 16, sixth form/college, undergraduate degree, postgraduate degree), marital status (married vs not married), employment status (working vs not working), partner's employment status (working vs not working) and whether the woman had previously been diagnosed with either anxiety or depression. Finally, listening to music was categorised as 'rarely; a couple of times a week; every day <1hr; every day 1-2hrs; every day 3-5hrs; every day 5+hrs'.

<u>Statistics</u>

Data were analysed using Stata v14. Multiple linear regression models were used to explore the effects of listening to music on wellbeing and PND. Model 1 was unadjusted, while Model 2 adjusted for baseline wellbeing/depression, mother's age, maternal education status, household income and number of previous children, as well as how many weeks the baby was post-birth, and the mother's marital status at T2, whether she was working at T1 or T2, whether her partner was working at T2, and previous histories of both anxiety and depression.

All models displayed linearity as assessed by augmented partial residual plots with lowess smoothing; multicollinearity as assessed by checking variance inflation factors; normality as assessed using kernel density plots, standardised normal probability (P-P) plots and Q-Q plots; and there was no evidence of outliers or undue influence as assessed using added variable plots regressing each variable against all others, through stem and leaf plots, and through assessing covariance ratios, Cook's distance and leverage. The wellbeing regression models demonstrated homoscedasticity as assessed by plotting the residuals versus fitted (predicted) values and using the Breusch-Pagan test for heteroskedasticity. However, the depression regression models showed signs of heteroskedasticity, so robust standard errors were calculated.

Planned sensitivity analyses were then conducted in order to ascertain whether listening to music was of particular value for (i) women with above or below average wellbeing during pregnancy (using the

For peer review only - http://bmjopen.bmj.com/site/about/guidelines.xhtml

For peer review only

NEF cut-offs) or different levels of depression during pregnancy (using EPDS>=10 cut-off) respectively; and (ii) women with or without histories of anxiety or depression.

In order to take account of potential demographic differences between those who provided data at Q3 and those who failed to, the propensity score for non-response was calculated and inverse probability weighting was applied to the T3 regression models.

Results

Demographics. Regarding music listening habits, just 5.6% of women reported listening to music 'rarely' in the final trimester of pregnancy. 17.2% reported listening just a 'couple of times a week'. The rest of women reported listening daily: 34.4% reported listening every day but for less than 1 hour; 29.9% reported listening every day for 1-2 hours; 8.6% reported listening every day for 3-5 hours; and 4.3% reported listening every day for 5 hours or more. While these analyses focused on quantity of music listening as a predictor, we did record genre of music listened to. 21.0% reported listening to jazz, 93.7% reported listening to pop music, 57.7% reported listening to rock music, 34.2% reported listening to classical music, 22.8% reported listening to folk music and 42.8% reported listening to R&B.

The average wellbeing score at T1 was 24.1 (SD=3.9, range 11.25-35), at T2 was 23.9 (SD=4.3, range 7-35) and at T3 was 23.8 (SD=4.1, range 7-35) (Table 1). In order to calculate the change in wellbeing amongst these women, we analysed the difference in scores from T1 to T2 and T1 to T3. 39.2% of mothers experienced a decrease or at least 1 point in their wellbeing from T1 to T2, while 30.4% experienced no change and 30.4% experienced an improvement of at least 1 point. This equated to a strong correlation between results at T1 and T2 ($r=0.63$, $p<.001$). From T1 to T3, 41.4% of mothers experienced a decrease or at least 1 point in their wellbeing, while 25.7% experienced no change and 32.9% experienced an improvement of at least 1 point. This equated to a strong correlation between results at T1 and T3 ($r=0.61$, $p<.001$).

Table 1: Levels of wellbeing and postnatal depression during pregnancy (T1), 0-3 months post-birth (T2) and 4-6 months post-birth (T3).

	T1	T2	T3
Wellbeing			
Poor (<22)	29.4%	31.1%	31.6%
Below average (22-24)	25.6%	24.1%	25.4%
Average (25-26)	23.5%	21.3%	20.9%
Good (27-28)	10.1%	11.7%	13.7%
Excellent (29-35)	11.4%	11.9%	8.5%
Depression			
EPDS<10	74.7%	72.2%	73.3%

For peer review only - http://bmjopen.bmj.com/site/about/guidelines.xhtml

EPDS>=10	25.3%	27.9%	26.7%

As with wellbeing, we calculated the change in symptoms of PND from T1 to T2 and T1 to T3 (table 1). 43.6% of mothers experienced an increase in the number of symptoms of PND they were experiencing from T1 to T2, while 11.2% experienced no change, and 45.3% of mothers experienced an improvement in symptoms. This equated to a strong correlation between results at T1 and T2 (r=0.58, p<.001). From T1 to T3, 56.4% of mothers experienced an increase in the number of symptoms of PND they were experiencing, while 11.6% experienced no change and 43.6% experienced an improvement in symptoms. This equated to a strong correlation between results at T1 and T3 (r=0.55, p<.001). In terms of the interaction between wellbeing and symptoms of PND, there was a large correlation between the two at T1 (r=-0.67, p<.001), T2 (r=-0.76, p<.001) and T3 (r=-0.77, p<.001).

Regression results: Listening to music while pregnant was associated with higher wellbeing levels 0-3 months post-birth, even when accounting for potential confounding variables (see Table 2). However, effects were not evident 4-6 months post-birth. There was also an association between listening to music while pregnant and symptoms of PND, even when accounting for potential confounding variables, with listening to music during pregnancy associated with lower symptoms of PND in the first 3 months post-birth. As with wellbeing, these results were no longer evident by months 4-6 post-birth.

	Wellbeing	Symptoms of PND

Table 2: Associations between listening to music during pregnancy on wellbeing and symptoms of postnatal depression post-birth

For peer review only - http://bmjopen.bmj.com/site/about/guidelines.xhtml

**Months 0-3 post birth**								
	β	SE	95% CI	t	β	SE	95% CI	t
Model 1	**0.63**	**0.19**	**0.26 – 1.00**	**3.38*** **	**-0.52**	**0.23**	**-0.98 – -0.06**	**-2.21***
	**R^2=0.03, F(1, 393)=11.44, p=.001**				**R^2=0.01, F(1,393)=4.74, p=.03**			
Model 2	**0.40**	**0.15**	**0.10 - 0.70**	**2.61****	**-0.39**	**0.19**	**-0.76 - -0.03**	**-2.10***
	**R^2=0.43, F(18,376)=15.97, p<.001**				**R^2=0.39, F(18,376)=9.58, p<.001**			
**Months 3-6 post birth**								
	β	SE	95% CI	t	β	SE	95% CI	t
Model 1	0.32	0.22	-0.11 – 0.76	1.47	-0.25	0.27	-0.78 – 0.28	-0.93
	R^2=0.01, F(1,305)=2.17, p=.14				R^2=0.003, F(1,301)=0.86, p=.35			
Model 2	0.20	0.16	-0.12 – 0.52	1.23	-0.18	0.21	-0.59 – 0.23	-0.86
	R^2=0.44, F(18,284)=11.22, p<.001				R^2=0.37, F(18,280)=7.19, p<.001			

*Model 1: unadjusted; Model 2: adjusted for baseline wellbeing/depression, mother's age, maternal education status, household income and number of previous children, as well as how many weeks the baby was post-birth, and the mother's marital status at T2, whether she was working at T1 or T2 and whether her partner was working at T2 and previous histories of both anxiety and depression. *p<.05, **p<.01, ***p<.001*

Further analyses: Sensitivity analyses of the wellbeing regression models revealed that women with poorer mental health during pregnancy had the potential to benefit most from listening to music. Listening to music was significantly associated with wellbeing post-birth amongst women with 'below average' and 'poor' levels of wellbeing (WEMWBS<25) at baseline (β=0.69, SE=0.21, 95%CI 0.29 to 1.10, p=.001) but not amongst those with 'average', 'good' or 'excellent' wellbeing at baseline (β=0.25, SE=0.28, 95%CI -0.30 to 0.79, p=.38). Sensitivity analyses of the PND regression models suggested similar results, with significant associations only found for women who were displaying symptoms of PND at baseline (EPDS≥10) (β=-1.75, SE=0.52, 95%CI -2.78 to -0.72, p=.001), but not for those who were not displaying symptoms (EPDS<10) (β=-0.26, SE=0.24, 95%CI -0.73 to 0.21, p=.27).

Similarly, sensitivity analyses also identified that those with previous histories of anxiety or depression were most likely to benefit. Significant associations found for wellbeing post-birth were found amongst both those who had a history of anxiety (β=1.10, SE=0.25, 95%CI 0.61 to 1.58, p<.001) or depression (β=1.07, SE=0.26, 95%CI 0.56 to 1.58, p<.001). Similarly, significant associations for symptoms of depression post-birth were found amongst both those who had a history of anxiety (β=-1.11, SE=0.36, 95%CI -1.82 to -0.41, p=.002) or depression (β=-0.87, SE=0.37, 95%CI -1.61 to -0.13, p=.021).

Finally, in order to try and ascertain whether listening to music led to changes in mental health or whether mental health led to changes in listening habits, we ran additional analyses reversing the variable order. There was no evidence that levels of wellbeing during pregnancy were associated with the likelihood of listening to music either 3 or 6 months post-birth. However, there was some

For peer review only - http://bmjopen.bmj.com/site/about/guidelines.xhtml

For peer review only

indication that depression symptoms in the final trimester of pregnancy were associated with listening habits 3 months post-birth (β=-0.03, SE=0.01, 95%CI -0.05 to -0.01, p=.003).

Discussion

This study explored associations between listening to music in the final trimester of pregnancy and mental health and wellbeing in mothers post-birth. Listening was found to be associated with higher levels of wellbeing and reduced symptoms of PND in the first 3 months post-birth, even when adjusting for baseline mental health and potential confounding variables. These results appear to be particularly found amongst women with lower levels of wellbeing at baseline. These findings echo the few existing studies in showing that listening to music is associated with better mental health in the perinatal period (30,31). However, to the authors' knowledge, this is the first study to show that listening to music during pregnancy is longitudinally associated with better mental health post-birth.

Across both symptoms of depression and wellbeing, however, associations were only found for the first 3 months post-partum, and had disappeared by the second quartile post-partum. The hypnosis study previously described also found results within the first 3 months post-partum (weeks 2 and 10) but did not measure beyond this, so there is little data available against which to benchmark these findings (16). Nevertheless, the immediate period post-birth has been highlighted as being of particular challenge for new mothers, with the transition into assuming maternal tasks and adjusting to the new role lasting until around the third month postpartum (13,34). It is possible, therefore, that any effects of music listening during the prenatal period are of most value during this transition period, but become less significant once mothers and their babies become more settled.

Of course, a key question is how listening to music is associated with better mental health and wellbeing in the postnatal period. There are a number of potential explanations. First, studies involving psychological tests, neuroimaging, biomarker analyses and ethnographic observations have shown that listening to music can have marked effects on stress and anxiety (25). Specifically in relation to pregnant women, listening to music for just 30 minutes can reduce cortisol levels and anxiety (29). Wider studies involving listening to music have shown it to be particularly effective at reducing psychological and physiological responses to stress, especially when people deliberately listen to music in order to help them relax (35,36). This effect of music on stress has in turn been linked specifically through to theories around wellbeing (25,37), with a wide literature linking stress and anxiety with both mental health and wellbeing (38,39). It is proposed that high levels of anxiety might hinder women's adaptation to motherhood in the initial postpartum period, with negative effects on wellbeing (40). Consequently, it is possible that the relaxing effects of listening to music during the pregnancy period helps to act as a buffer for feelings of stress and anxiety, thereby supporting mothers in maintaining their adaptation and leading to enhanced wellbeing.

For peer review only - http://bmjopen.bmj.com/site/about/guidelines.xhtml

Another potential explanation relates to the effects of music on mood. Mood regulation has been identified as one of the prime reasons why people listen to music, with models of mood regulation by music highlighting its effects on mood-related subjective experience (including the intensity and clarity of moods), physiological responses (such as energy levels and movement) and behaviours (such as their ability to express emotions) (41). Music listening has been found to modulate depression and wellbeing (42,43). Early low mood during the prenatal period is directly associated with lower wellbeing and postnatal depression post-birth (44), leading to propositions that interventions that deliberately attempt to cultivate positive emotions, such as relaxation therapies and interventions focused on finding positive meaning, could directly optimise health and wellbeing in this population. Consequently, it is possible that another route by which listening to music in the third trimester of pregnancy is associated with improvements in mental health and wellbeing is via enhancing mood.

Finally, a third explanation is that listening to music in itself did not have an effect post-birth but did enhance coping skills in women while they were still pregnant, which in turn led to higher wellbeing post-birth. Music listening has been linked with both problem-oriented coping and emotion-oriented coping, specifically with results showing that problem-oriented coping by music listening in women is linked to lower depression levels (45). Life transitions (such as the perinatal period) depend on both health and wellbeing and also on appraisal and coping responses. In the hypnosis study previously mentioned, the authors proposed that the intervention during pregnancy helped mothers to maintain and enhance their wellbeing whilst pregnant, which in turn influenced their appraisal of the perinatal transition period and supported their coping responses (16). It is possible that a similar process took place through listening to music, with listening to music supporting coping in the prenatal period, which encouraged mothers' own coping skills, which in turn led to better coping during and post-birth and consequent higher wellbeing. Indeed, even just in relation to the birth, a number of birth preparation courses focus on relaxation and mood optimisation, which have been shown to lead to less negative affect and better coping during labour and delivery (46). Given that a significant predictor of PND is the birth experience, enhanced coping prior to the birth, perhaps through music listening, could be an important factor in postnatal wellbeing (47).

This study is limited by a few factors. First, the study followed a cohort of women rather than being interventional, so it is not possible to confirm causality. However, the study had a longitudinal design, there was no evidence of reverse causality in relation to wellbeing, there are plausible proposed mechanistic explanations, and there is a strong body of previous literature causally linking music and mental health in other populations. So this study provides promising preliminary evidence that remains to be tested in a future experimental design. A second limitation is that the population in this

For peer review only - http://bmjopen.bmj.com/site/about/guidelines.xhtml

study was not nationally representative. Nevertheless, there was a clear spread of participants from varying socio-economic backgrounds as well as variations in the levels of exposure and outcome variables. So the dataset provides interesting and suitable preliminary data on the longitudinal associations between music listening and mental health shown here. Third, this study explored the impact of all music listening, not specifying particular genres. Previous research has suggested that certain genres of music (or more specifically compositional aspects of music such as its valence and arousal levels) can lead to different responses, such as variations in relaxation or mood (48). However, most of these genre-specific effects have been found in tightly-controlled lab-based studies, and literature from real-world interaction with music has suggested that musical preference might be more important in determining the effects of music (49). This study followed these real-world studies in recording what genres people did listen to but measuring the quantity of listening based on preference rather than genre. Future studies could explore the impact of different genres on mental health in the perinatal period.

In conclusion, this study provides the first preliminary evidence that listening to music during the third trimester of pregnancy could be protective against symptoms of PND and low wellbeing in the first three months post-birth. Music listening is an attractive intervention in that it is readily available to people from all echelons of society regardless of socioeconomic status, educational attainment or cultural background. It can be carried out in a range of contexts so is not restricted to particular places or times. It is also inexpensive: indeed the majority of women in the Western countries have access to recorded music already. Finally, there are no obvious side effects from listening to music. Consequently, listening to music could be recommended as a way of supporting pregnant women; in particular those who demonstrate low wellbeing or symptoms of PND.

Authors' contributions

DF and RP designed the study and collected data. DF ran the analyses and drafted the paper. Both authors critically revised the manuscript and approved it for submission.

For peer review only

For peer review only - http://bmjopen.bmj.com/site/about/guidelines.xhtml

References

1. Khalifeh H, Brauer R, Toulmin H, Howard LM. Perinatal mental health: What every neonatologist should know. Early Hum Dev. 2015 Nov 1;91(11):649–53.
2. Morrell C, Warner R, Slade P, Dixon S, Walters S, Paley G, et al. Psychological interventions for postnatal depression: cluster randomised trial and economic evaluation. The PoNDER trial. Health Technol Assess [Internet]. 2009 Jun [cited 2016 Jul 26];13(30). Available from: http://www.journalslibrary.nihr.ac.uk/hta/volume-13/issue-30
3. Cooper PJ, Murray L. Postnatal depression. BMJ. 1998 Jun 20;316(7148):1884–6.
4. Junge C, Garthus-Niegel S, Slinning K, Polte C, Simonsen TB, Eberhard-Gran M. The Impact of Perinatal Depression on Children's Social-Emotional Development: A Longitudinal Study. Matern Child Health J. 2017 Mar 1;21(3):607–15.
5. Green JM, Kafetsios K. Positive experiences of early motherhood: Predictive variables from a longitudinal study. J Reprod Infant Psychol. 1997 May 1;15(2):141–57.
6. Keyes CL. Mental Illness and/or Mental Health? Investigating Axioms of the Complete State Model of Health. J Consult Clin Psychol. 2005;73(3):539–48.
7. Boyce PM, Johnstone SJ, Hickey AR, Morris-Yates AD, Harris MG, Strachan T. Functioning and well-being at 24 weeks postpartum of women with postnatal depression. Arch Womens Ment Health. 2000 Dec 11;3(3):91–7.
8. Jomeen J. The importance of assessing psychological status during pregnancy, childbirth and the postnatal period as a multidimensional construct: A literature review. Clin Eff Nurs. 2004 Sep;8(3–4):143–55.
9. Allan C, Carrick-Sen D, Martin CR. What is perinatal well-being? A concept analysis and review of the literature: J Reprod Infant Psychol [Internet]. 2013 [cited 2017 Jun 8];31(4). Available from: http://www.tandfonline.com/doi/abs/10.1080/02646838.2013.791920
10. Beck CT. Predictors of postpartum depression: an update. Nurs Res. 2001 Oct;50(5):275–85.
11. Milgrom J, Gemmill AW, Bilszta JL, Hayes B, Barnett B, Brooks J, et al. Antenatal risk factors for postnatal depression: a large prospective study. J Affect Disord. 2008 May;108(1–2):147–57.
12. Robertson E, Grace S, Wallington T, Stewart DE. Antenatal risk factors for postpartum depression: a synthesis of recent literature. Gen Hosp Psychiatry. 2004 Jul;26(4):289–95.
13. Leifer M. Psychological effects of motherhood : a study of first pregnancy / Myra Leifer. New York: Praeger; 1980.
14. Elliott SA, Leverton TJ, Sanjack M, Turner H, Cowmeadow P, Hopkins J, et al. Promoting mental health after childbirth: a controlled trial of primary prevention of postnatal depression. Br J Clin Psychol. 2000 Sep;39 (Pt 3):223–41.
15. Spinelli MG. Prevention of postpartum mood disorders. In: Postpartum mood disorders. Arlington, VA, US: American Psychiatric Association; 1999. p. 217–35.
16. Guse T, Wissing M, Hartman W. The effect of a prenatal hypnotherapeutic programme on postnatal maternal psychological well□being. J Reprod Infant Psychol. 2006 May;24(2):163–77.
17. Chan MF, Wong ZY, Thayala NV. The effectiveness of music listening in reducing depressive symptoms in adults: A systematic review. Complement Ther Med. 2011 Dec 1;19(6):332–48.
18. Cassidy JW, Standley JM. The Effect of Music Listening on Physiological Responses of Premature Infants in the NICU. J Music Ther. 1995 Dec 21;32(4):208–27.
19. Pelletier CL. The effect of music on decreasing arousal due to stress: a meta-analysis. J Music Ther. 2004 Fall;41(3):192–214.
20. Siedliecki SL, Good M. Effect of music on power, pain, depression and disability. J Adv Nurs. 2006 Jun 1;54(5):553–62.

For peer review only - http://bmjopen.bmj.com/site/about/guidelines.xhtml

21. Hsu W-C, Lai H-L. Effects of music on major depression in psychiatric inpatients. Arch Psychiatr Nurs. 2004 Oct 1;18(5):193–9.
22. Chan MF, Chan EA, Mok E, Kwan Tse FY. Effect of music on depression levels and physiological responses in community-based older adults. Int J Ment Health Nurs. 2009 Aug 1;18(4):285–94.
23. Laukka P. Uses of music and psychological well-being among the elderly. J Happiness Stud. 2007 Jun 1;8(2):215.
24. Haake AB. Individual music listening in workplace settings An exploratory survey of offices in the UK. Music Sci. 2011 Mar 1;15(1):107–29.
25. Vastfjall D, Juslin PN, Hartig T. Music, subjective wellbeing, and health: the role of everyday emotions. In: MacDonald R, Kreutz G, Mitchell L, editors. Music, Health, and Wellbeing. Oxford University Press; 2013.
26. McCaffrey R. Music Listening: Its Effects in Creating a Healing Environment. J Psychosoc Nurs Ment Health Serv. 2008 Oct 1;46(10):39–44.
27. Batt-Rawden KB, DeNora T, Ruud E. Music Listening and Empowerment in Health Promotion: A Study of the Role and Significance of Music in Everyday Life of the Long-term Ill. Nord J Music Ther. 2005 Jul 1;14(2):120–36.
28. Skånland MS. Everyday music listening and affect regulation: The role of MP3 players. Int J Qual Stud Health Well-Being [Internet]. 2013 [cited 2017 Jun 14];8. Available from: /pmcc/articles/PMC3740499/?report=abstract
29. Ventura T, Gomes MC, Carreira T. Cortisol and anxiety response to a relaxing intervention on pregnant women awaiting amniocentesis. Psychoneuroendocrinology. 2012 Jan;37(1):148–56.
30. Nwebube C, Glover V, Stewart L. Prenatal listening to songs composed for pregnancy and symptoms of anxiety and depression: a pilot study. BMC Complement Altern Med. 2017 May 8;17(1):256.
31. Fancourt D, Perkins R. Associations between singing to babies and symptoms of postnatal depression, wellbeing, self-esteem and mother-infant bond. Public Health. 2017 Apr 1;145:149–52.
32. Cox JL, Holden JM, Sagovsky R. Detection of postnatal depression. Development of the 10-item Edinburgh Postnatal Depression Scale. Br J Psychiatry. 1987 Jun 1;150(6):782–6.
33. Stewart-Brown S, Tennant A, Tennant R, Platt S, Parkinson J, Weich S. Internal construct validity of the Warwick-Edinburgh Mental Well-being Scale (WEMWBS): a Rasch analysis using data from the Scottish Health Education Population Survey. Health Qual Life Outcomes. 2009;7:15.
34. Pridham KF, Chang AS. Transition to being the mother of a new infant in the first 3 months: maternal problem solving and self-appraisals. J Adv Nurs. 1992 Feb;17(2):204–16.
35. Krout RE. The effects of single-session music therapy interventions on the observed and self-reported levels of pain control, physical comfort, and relaxation of hospice patients. Am J Hosp Palliat Med. 2001 Dec;18(6):383–90.
36. Linnemann A, Strahler J, Nater UM. The stress-reducing effect of music listening varies depending on the social context. Psychoneuroendocrinology. 2016 Oct 1;72:97–105.
37. Krout RE. Music listening to facilitate relaxation and promote wellness: Integrated aspects of our neurophysiological responses to music. Arts Psychother. 2007;34(2):134–41.
38. Iwata M, Ota KT, Duman RS. The inflammasome: pathways linking psychological stress, depression, and systemic illnesses. Brain Behav Immun. 2013 Jul;31:105–14.
39. Vedhara K, Miles J, Bennett P, Plummer S, Tallon D, Brooks E, et al. An investigation into the relationship between salivary cortisol, stress, anxiety and depression. Biol Psychol. 2003 Feb;62(2):89–96.
40. Pond E, Kemp V. A comparison between adolescent and adult women on prenatal anxiety and self-confidence. Matern Child Nurs J. 1992;20(1):11–20.

For peer review only - http://bmjopen.bmj.com/site/about/guidelines.xhtml

41. Saarikallio S, Erkkilä J. The role of music in adolescents' mood regulation. Psychol Music. 2007 Jan 1;35(1):88–109.
42. Chin T, Rickard NS. Emotion regulation strategy mediates both positive and negative relationships between music uses and well-being. Psychol Music. 2014 Sep 1;42(5):692–713.
43. Lai Y-M. Effects of Music Listening on Depressed Women in Taiwan. Issues Ment Health Nurs. 1999 Jan 1;20(3):229–46.
44. Fleming A, N. Ruble D, L. Flett G, L. Shaul D. Postpartum Adjustment in First-Time Mothers: Relations Between Mood, Maternal Attitudes, and Mother-Infant Interactions. Dev Psychol. 1988 Jan 1;24:71–81.
45. Miranda D, Claes M. Music listening, coping, peer affiliation and depression in adolescence. Psychol Music. 2009 Apr 1;37(2):215–33.
46. Duncan S, J. Markman H. Intervention programs for the transition to parenthood: Current status from a prevention perspective. 2017 Oct 26;
47. Green JM. Expectations and experiences of pain in labor: findings from a large prospective study. Birth Berkeley Calif. 1993 Jun;20(2):65–72.
48. Labbe E, Schmidt N, Babin J, Pharr M. Coping with stress: the effectiveness of different types of music. Appl Psychophysiol Biofeedback. 2007 Dec;32(3–4):163–8.
49. Fancourt D, Ockelford A, Belai A. The psychoneuroimmunological effects of music: A systematic review and a new model. Brain Behav Immun. 2014 Feb;36:15–26.

For peer review only

STROBE Statement—Checklist of items that should be included in reports of ***cohort studies***

	Item No	Recommendation	Page
**Title and abstract**	1	(*a*) Indicate the study's design with a commonly used term in the title or the abstract	1
		(*b*) Provide in the abstract an informative and balanced summary of what was done and what was found	2
**Introduction**			
Background/rationale	2	Explain the scientific background and rationale for the investigation being reported	4-5
Objectives	3	State specific objectives, including any prespecified hypotheses	5
**Methods**			
Study design	4	Present key elements of study design early in the paper	6
Setting	5	Describe the setting, locations, and relevant dates, including periods of recruitment, exposure, follow-up, and data collection	6
Participants	6	(*a*) Give the eligibility criteria, and the sources and methods of selection of participants. Describe methods of follow-up	6
		(*b*) For matched studies, give matching criteria and number of exposed and unexposed	
Variables	7	Clearly define all outcomes, exposures, predictors, potential confounders, and effect modifiers. Give diagnostic criteria, if applicable	6-7
Data sources/ measurement	8*	For each variable of interest, give sources of data and details of methods of assessment (measurement). Describe comparability of assessment methods if there is more than one group	6-7
Bias	9	Describe any efforts to address potential sources of bias	7-8
Study size	10	Explain how the study size was arrived at	
Quantitative variables	11	Explain how quantitative variables were handled in the analyses. If applicable, describe which groupings were chosen and why	6-7
Statistical methods	12	(*a*) Describe all statistical methods, including those used to control for confounding	7
		(*b*) Describe any methods used to examine subgroups and interactions	7-8
		(*c*) Explain how missing data were addressed	8
		(*d*) If applicable, explain how loss to follow-up was addressed	8
		(*e*) Describe any sensitivity analyses	7-8
**Results**			
Participants	13*	(a) Report numbers of individuals at each stage of study—eg numbers potentially eligible, examined for eligibility, confirmed eligible, included in the study, completing follow-up, and analysed	6
		(b) Give reasons for non-participation at each stage	
		(c) Consider use of a flow diagram	
Descriptive data	14*	(a) Give characteristics of study participants (eg demographic, clinical, social) and information on exposures and potential confounders	6
		(b) Indicate number of participants with missing data for each variable of interest	N/A
		(c) Summarise follow-up time (eg, average and total amount)	6
Outcome data	15*	Report numbers of outcome events or summary measures over time	8-9
Main results	16	(*a*) Give unadjusted estimates and, if applicable, confounder-adjusted estimates and their precision (eg, 95% confidence interval). Make clear which	8-9

For peer review only

For peer review only - http://bmjopen.bmj.com/site/about/guidelines.xhtml

		confounders were adjusted for and why they were included	
		(*b*) Report category boundaries when continuous variables were categorized	
		(*c*) If relevant, consider translating estimates of relative risk into absolute risk for a meaningful time period	
Other analyses	17	Report other analyses done—eg analyses of subgroups and interactions, and sensitivity analyses	9-10
**Discussion**			
Key results	18	Summarise key results with reference to study objectives	10
Limitations	19	Discuss limitations of the study, taking into account sources of potential bias or imprecision. Discuss both direction and magnitude of any potential bias	12
Interpretation	20	Give a cautious overall interpretation of results considering objectives, limitations, multiplicity of analyses, results from similar studies, and other relevant evidence	12
Generalisability	21	Discuss the generalisability (external validity) of the study results	12
**Other information**			
Funding	22	Give the source of funding and the role of the funders for the present study and, if applicable, for the original study on which the present article is based	3

*Give information separately for exposed and unexposed groups.

Note: An Explanation and Elaboration article discusses each checklist item and gives methodological background and published examples of transparent reporting. The STROBE checklist is best used in conjunction with this article (freely available on the Web sites of PLoS Medicine at http://www.plosmedicine.org/, Annals of Internal Medicine at http://www.annals.org/, and Epidemiology at http://www.epidem.com/). Information on the STROBE Initiative is available at http://www.strobe-statement.org.

For peer review only

Could listening to music during pregnancy be protective against postnatal depression and poor wellbeing post-birth? Longitudinal associations from a preliminary prospective cohort study.

Journal:	*BMJ Open*
Manuscript ID	bmjopen-2017-021251.R1
Article Type:	Research
Date Submitted by the Author:	16-Mar-2018
Complete List of Authors:	Fancourt, Daisy; University College London Research Department of Epidemiology and Public Health, Department of Behavioural Science and Health<br>Perkins, Rosie; Royal College of Music, Centre for Performance Science; Imperial College London, Faculty of Medicine
**Primary Subject Heading**:	Mental health
Secondary Subject Heading:	Obstetrics and gynaecology
Keywords:	MENTAL HEALTH, Depression & mood disorders < PSYCHIATRY, Maternal medicine < OBSTETRICS

SCHOLARONE™ Manuscripts

For peer review only

Could listening to music during pregnancy be protective against postnatal depression and poor wellbeing post-birth? Longitudinal associations from a preliminary prospective cohort study.

Daisy Fancourt PhD [a,b] and Rosie Perkins PhD [b,c]*

[a] Department of Behavioural Science and Health, University College London, London WC1E 7HB

[b] Faculty of Medicine, Imperial College London, London SW7 2AZ

[c] Centre for Performance Science, Royal College of Music, London SW7 2BS

* Corresponding author: Dr Daisy Fancourt, 1-19 Torrington Place, London, WC1E 7HB, d.fancourt@ucl.ac.uk

For peer review only

Abstract

Objectives: This study explored whether listening to music during pregnancy is longitudinally associated with lower symptoms of postnatal depression and higher wellbeing in mothers post-birth.

Design: Prospective cohort study.

Participants: We analysed data from 395 new mothers aged over 18 who provided data in the third trimester of pregnancy and 3 and 6 months later (0-3 and 4-6 months post birth).

Primary and secondary outcome measures: Postnatal depression was measured using the Edinburgh Postnatal Depression Scale (EPDS), and wellbeing was measured using the Short Warwick-Edinburgh Mental Wellbeing Scale (SWEMWBS). Our exposure was listening to music and was categorised as 'rarely; a couple of times a week; every day <1hr; every day 1-2hrs; every day 3-5hrs; every day 5+hrs'. Multivariable linear regression analyses were carried out to explore the effects of listening to music during pregnancy on depression and wellbeing post birth, adjusted for baseline mental health and potential confounding variables.

Results: Listening during pregnancy is associated with higher levels of wellbeing (β=0.40, SE=0.15, 95%CI 0.10 to 0.70) and reduced symptoms of postnatal depression (β=-0.39, SE=0.19, 95%CI -0.76 to -0.03) in the first 3 months post-birth. However, effects disappear by 4-6 months post-birth. These results appear to be particularly found amongst women with lower levels of wellbeing and high levels of depression at baseline.

Conclusions: Listening to music could be recommended as a way of supporting mental health and wellbeing in pregnant women; in particular those who demonstrate low wellbeing or symptoms of postnatal depression.

Keywords

Postnatal depression, wellbeing, mental health, music, perinatal

Strengths and limitations of this study

- This preliminary prospective cohort study tracked a sample of women across the perinatal period providing data at 12 week intervals.
- The data include a rich set of variables on music listening behaviours amongst participants.

For peer review only - http://bmjopen.bmj.com/site/about/guidelines.xhtml

- We adjusted for all identified confounding variables in our analyses and ran sensitivity analyses to test our assumptions.
- The data are not nationally representative, although there is a clear spread of participants from varying socio-economic backgrounds as well as variations in the levels of exposure and outcome variables.
- As this is a cohort study and not interventional, it is not possible to confirm causality.

Funding statement

The study was funded by Arts Council England Research Grants Fund, grant number 29230014 (Lottery). D.F. is supported by the Wellcome Trust [205407/Z/16/Z]. The study was approved by the NHS REC (15/SS/016). The study team acknowledge the support of the National Institute of Health Research Clinical Research Network (NIHR CRN). The authors would like to thank the hospitals involved as Participant Identification Centres as well as Miss Sunita Sharma, Prof Aaron Williamon and Sarah Yorke for their support with the study.

Competing interests statement

Both authors declare no conflict of interest.

For peer review only

Introduction

Perinatal mental health problems affect around 20% of women at some point during the perinatal period (1). In terms of conditions characterised by negative symptomology, postnatal depression (PND) is one of the most common problems, and is a debilitating condition with symptoms including fatigue, irritability, insomnia and anhedonia; symptoms which in 25% of affected women last for at least one year (2). Over the last two decades, there has been significant research into the effects of PND on mother and infant as well as attention paid to how it can be prevented or managed (3,4). However, in terms of conditions relating more to the absence of positive symptomology, such as low hedonic or eudemonic wellbeing, there has been much less research. The few studies that do exist have found that negative mood, as indicated by the Edinburgh Postnatal Depression Scale (EPDS), has a correlation of just -0.46 with positive experiences of motherhood, as indicated by a principal component analysis of six experiences of motherhood (POSMO) (5). This suggests that, as in the wider population, depression and wellbeing are separate constructs in the context of perinatal mental health and a positive perinatal experience is more than simply the inversion of negative mood (6). Building on this, women even without PND have been found to demonstrate impairments in emotional problems and vitality, suggesting that even in the absence of depression mothers can have impaired wellbeing (7). In light of this, a review has argued that psychological wellbeing defined as a multidimensional construct should be an integral part of maternity care (8), and a more recent construct analysis has highlighted the importance not just of identifying PND but also of identifying women with sub-optimal perinatal wellbeing and supporting them to achieve positive psychological functioning (9).

In seeking to support the perinatal mental health of women, the pregnancy period has been highlighted as critical. Prenatal mental health has repeatedly been shown to be one of the largest predictors of postnatal depression (10–12) and wellbeing (9). In particular, the third trimester of pregnancy has been identified as an important transition period involving adaptation to emotional and physical changes, leading to feelings of wellbeing often less pronounced than in the previous trimesters (13). Early detection of symptoms of depression and low wellbeing during pregnancy and prompt intervention is therefore important in reducing adverse consequences.

In light of this, there are a number of interventions that have been developed to try and support mental health in the prenatal period as a way of reducing postnatal mental health problems, in particular focusing on the third trimester as a point of intervention. There have been findings that support the application of cognitive-behavioural and interpersonal psychotherapy, suggesting that depression following childbirth could be prevented by brief interventions in the prenatal period (14,15). In exploring other interventions, prenatal hypnotherapy has been found to significantly reduce PND and improve psychological wellbeing at two weeks and ten weeks postpartum (16). And a psychosocial

For peer review only - http://bmjopen.bmj.com/site/about/guidelines.xhtml

intervention involving group meetings to discuss aspects of parenthood in the final trimester of pregnancy and first 6 months postpartum has been found to reduce PND amongst first-time mothers (14). However, as many mothers continue to work full-time until shortly before their due dates, in-person interventions may not be feasible for all mothers and are of course limited by what is available in different geographical areas. As a result, there is a need to identify other home-based interventions that could provide similar mental health support.

Over the past two decades, there has been increasing research showing the effects of listening to music on mental health. A number of reviews have demonstrated the effects of regular music listening including in enhancing mental health in the general population (17), reducing distress in premature infants (18), and reducing stress in adults (19). Specifically in relation to depression, listening to music has been shown to reduce depression amongst adults with chronic pain (20), psychiatric inpatients (21), and older adults (17,22). In relation to wellbeing, music listening has been shown to be associated with better wellbeing not just in controlled interventions but also as a result of ordinary day-to-day listening. A Swedish study involving 500 older adults found associations between music listening and wellbeing, even when controlling for potential confounding variables (23). Studies tracking daily activities have linked music listening with enhanced wellbeing both in the workplace and in the wider context of people's lives (24,25). Further, music has also been shown to contribute to creating supportive healthy environments, connecting individuals with their emotions and promoting wellbeing (26). Finally, theoretical studies have highlighted the role of music listening in enhancing affect, wellness and resources for recovery and quality of life (27,28). Consequently, both directed music listening interventions and routine day-to-day music listening can affect levels of depression and wellbeing in a range of different populations.

Specifically in relation to the perinatal period, a few studies have suggested that music listening may be supportive for mental health. Listening to music for just 30 minutes has been found to reduce cortisol levels and anxiety in pregnant women, leading to recommendations that pregnant women might benefit from regular listening to music as a practice of relaxation (although the effects of regular listening were not tested) (29). A recent study found that women who listened to recorded music for 20 minutes a day for 12 weeks during their pregnancy had significant improvements in anxiety and depression (30). However, the study did not track outcomes postnatally and involved a small sample of women. And a further study has found cross-sectional associations between listening to music and depression and wellbeing amongst new mothers, with more frequent listening associated with better mental health (31). However, this study did not look longitudinally nor involve pregnant women.

For peer review only - http://bmjopen.bmj.com/site/about/guidelines.xhtml

Therefore, to date, despite promising results suggesting that listening to music can modulate mental health and wellbeing during the perinatal period, no studies have looked specifically at the impact of listening to music during pregnancy on depression and wellbeing post-birth. In order to address this research gap, this study tracked a cohort of mothers across the perinatal period in order to ascertain whether there was a relationship between music listening during pregnancy and postnatal mental health.

Methods

Participants and procedure

This study used data collected as part of a larger study exploring the impact of creative interventions on perinatal mental health. Women living in England in the last trimester of pregnancy (28 weeks or more) and the first nine months post birth (up to 40 weeks) were recruited from hospitals, General Practices, mother and baby charities and through social media in England across October 2015 to March 2016, and completed an anonymous cross-sectional online questionnaire. Women in the final trimester of pregnancy (28 weeks or more) were then invited to continue their participation in a longitudinal study. This involved providing another wave of data 3 months, 6 months and 9 months following the first date of data collection (which equated to providing baseline data T1 during pregnancy, T2 data in the first 3 months post-birth, T3 data in months 4-6 post-birth and T4 data in months 7-9 post-birth). From an initial sample of 550 mothers who consented to be involved in this longitudinal study, a total of 458 mothers provided T2 data (83%), 417 provided T3 data (75.8%) and 392 (71.3%) provided T4 data. The study received ethical approval from the UK NHS Research Ethics Service and all participants gave informed consent prior to involvement in the research.

For this study, and in light of the literature review presented above, we hypothesised that listening during pregnancy would support wellbeing and reduce symptoms of PND in the first trimester post-birth. We therefore focused on women in the longitudinal study who had provided complete data on the variables we selected for analyses at both T1 and T2: 395 women. However, we also ran some exploratory follow-up analyses with women who had also provided complete data at T3 (n=307) in order to explore if effects were maintained.

Measures

Symptoms of PND were measured using the Edinburgh Postnatal Depression Scale (EPDS), a 10-item scale used extensively both with pregnant women and new mothers, scored from 0-30 with 10+ indicative of possible symptoms of depression and higher scores of 13+ indicating more severe depression (32).

For peer review only - http://bmjopen.bmj.com/site/about/guidelines.xhtml

Wellbeing was measured using the Short Warwick-Edinburgh Mental Wellbeing Scale (SWEMWBS), a scale that encompasses both hedonic and eudemonic wellbeing comprising 7 items scored from 7 to 35 with higher scores representing higher levels of wellbeing. The raw scores were logit-transformed prior to analysis (33). The New Economics Foundation (NEF) suggests five levels of wellbeing based on quintile analyses of data in the UK Understanding Society Survey, 2009: poor (<22), below average (22-24), average (25-26), good (27-28) and excellent (29-35).

In addition, demographic variables assessed the women's number of weeks pregnant/post-birth, number of other children (0, 1, 2, 3 and 4+), household income (<£16,000, £16,000-£30,000, £31,000-£60,000, £61,000-£90,000, >£90,000), educational attainment (school to 16, sixth form/college, undergraduate degree, postgraduate degree), marital status (married vs not married), employment status (working vs not working), partner's employment status (working vs not working) and whether the woman had previously been diagnosed with either anxiety or depression.

Listening to music was categorised as 'rarely; a couple of times a week; every day <1hr; every day 1-2hrs; every day 3-5hrs; every day 5+hrs'.

<u>Statistics</u>

Data were analysed using Stata v14. Multivariable linear regression models were used to explore the effects of listening to music on wellbeing and PND. Frequency of listening to music had a normal distribution so was treated as a 6-point linear variable, with higher score indicating more frequent listening. For wellbeing and PND, we used raw scores. Model 1 was unadjusted, while Model 2 adjusted for baseline wellbeing/depression, mother's age, maternal education status, household income and number of previous children, as well as how many weeks the baby was post-birth, and the mother's marital status at T2, whether she was working at T1 or T2, whether her partner was working at T2, and previous histories of both anxiety and depression.

All models displayed linearity as assessed by augmented partial residual plots with lowess smoothing; multicollinearity as assessed by checking variance inflation factors; normality as assessed using kernel density plots, standardised normal probability (P-P) plots and Q-Q plots; and there was no evidence of outliers or undue influence as assessed using added variable plots regressing each variable against all others, through stem and leaf plots, and through assessing covariance ratios, Cook's distance and leverage. The wellbeing regression models demonstrated homoscedasticity as assessed by plotting the residuals versus fitted (predicted) values and using the Breusch-Pagan test for heteroskedasticity. However, the depression regression models showed signs of heteroskedasticity, so robust standard errors were calculated.

For peer review only - http://bmjopen.bmj.com/site/about/guidelines.xhtml

Planned sensitivity analyses were then conducted in order to ascertain whether baseline mental health during pregnancy was a moderator of the association between listening to music and mental health post birth. For this, we included an interaction term of Q1 mental health and Q1 listening habits in our regression models and then plotted two-way contour graphs to visualise the interaction (see Supplementary Figure 1).

Although there were no significant demographic differences between those who did and did not provide data at Q3, we wanted to take account of potential minor demographic differences between those who provided data at Q3 and those who failed to. So the propensity score for non-response was calculated using the indicators listed in Model 2 above (none of which significantly predicted missingness) and inverse probability weighting was applied to the T3 regression models. We confirmed goodness of fit using the Hosmer-Lemeshow test. Weighted analyses did not differ from unweighted analyses.

Results

Demographics. At T1, women had an average age of 31.9 years (SD=4.9, range 18-47) and an average of 32.9 weeks pregnant (SD=4.1, range 28-42). A total of 69.3% were married, 25.9% were cohabiting with a partner, a further 3.8% were in a relationship but not living together and 1% were single. Of those in relationships, 97.02% reported that their partners worked. Amongst the women, 13.2% had a basic education (equivalent to leaving school at 16), 16.5% had finished education at 18, 41.3% had an undergraduate degree and 29.1% had a postgraduate degree. Just 6.6% reported a household income of less than £16,000, 11.1% reported an income of £16,000-£30,000, 52.9% reported an income of £31,000 to £60,000, 17.5% reported an income of £61,000-90,000 and 11.9% reported an income of over £91,000.

Regarding music listening habits, just 5.6% of women reported listening to music 'rarely' in the final trimester of pregnancy. 17.2% reported listening just a 'couple of times a week'. The rest of women reported listening daily: 34.4% reported listening every day but for less than 1 hour; 29.9% reported listening every day for 1-2 hours; 8.6% reported listening every day for 3-5 hours; and 4.3% reported listening every day for 5 hours or more. While these analyses focused on quantity of music listening as a predictor, we did record genre of music listened to. 21.0% reported listening to jazz, 93.7% reported listening to pop music, 57.7% reported listening to rock music, 34.2% reported listening to classical music, 22.8% reported listening to folk music and 42.8% reported listening to R&B.

The average wellbeing score at T1 was 24.1 (SD=3.9, range 11.25-35), at T2 was 23.9 (SD=4.3, range 7-35) and at T3 was 23.8 (SD=4.1, range 7-35) (Table 1). In order to calculate the change in wellbeing amongst these women, we analysed the difference in scores from T1 to T2 and T1 to T3.

For peer review only - http://bmjopen.bmj.com/site/about/guidelines.xhtml

39.2% of mothers experienced a decrease or at least 1 point in their wellbeing from T1 to T2, while 30.4% experienced no change and 30.4% experienced an improvement of at least 1 point. From T1 to T3, 41.4% of mothers experienced a decrease or at least 1 point in their wellbeing, while 25.7% experienced no change and 32.9% experienced an improvement of at least 1 point.

Table 1: Levels of wellbeing and postnatal depression during pregnancy (T1), 0-3 months post-birth (T2) and 4-6 months post-birth (T3).

	T1	T2	T3
Wellbeing			
Poor (<22)	29.4%	31.1%	31.6%
Below average (22-24)	25.6%	24.1%	25.4%
Average (25-26)	23.5%	21.3%	20.9%
Good (27-28)	10.1%	11.7%	13.7%
Excellent (29-35)	11.4%	11.9%	8.5%
Depression			
EPDS<10	74.7%	72.2%	73.3%
EPDS>=10	25.3%	27.9%	26.7%

As with wellbeing, we calculated the change in symptoms of PND from T1 to T2 and T1 to T3 (table 1). 43.6% of mothers experienced an increase in the number of symptoms of PND they were experiencing from T1 to T2, while 11.2% experienced no change, and 45.3% of mothers experienced an improvement in symptoms. From T1 to T3, 56.4% of mothers experienced an increase in the number of symptoms of PND they were experiencing, while 11.6% experienced no change and 43.6% experienced an improvement in symptoms. In terms of the interaction between wellbeing and symptoms of PND, there was a large correlation between the two at T1 ($r=-0.67$, $p<.001$), T2 ($r=-0.76$, $p<.001$) and T3 ($r=-0.77$, $p<.001$).

Regression results: Listening to music while pregnant was associated with higher raw wellbeing scores 0-3 months post-birth, even when accounting for potential confounding variables, with greater frequency associated with greater effects (B=0.40, SE=0.15, 95%CI 0.10 to 0.70) (see Table 2). However, our exploratory analyses showed that effects were not evident 4-6 months post-birth. There was also an association between listening to music while pregnant and raw scores of symptoms of PND, even when accounting for potential confounding variables, with more frequent listening to music during pregnancy associated with lower symptoms of PND in the first 3 months post-birth (B=-0.39, SE=0.19, 95%CI -0.76 to -0.03). As with wellbeing, these results were no longer evident by months 4-6 post-birth.

Table 2: Associations between listening to music during pregnancy on wellbeing and symptoms of postnatal depression post-birth

For peer review only - http://bmjopen.bmj.com/site/about/guidelines.xhtml

Further analyses: Sensitivity analyses of the wellbeing regression models explored the potential moderating effect of mental health during pregnancy. Our analyses showed there was a significant interaction between listening and baseline wellbeing (B=-0.10 SE=0.04, 95%CI -0.17 to -0.02), with a two-way contour graph showing that listening to music particularly seemed to support those with lower wellbeing at baseline (see Supplementary Figure 1A). There was also a significant interaction

	Wellbeing				Symptoms of PND			
**Months 0-3 post birth**								
	B	SE	95% CI	t	B	SE	95% CI	t
Model 1	**0.63**	**0.19**	**0.26 – 1.00**	**3.38*** **	**-0.52**	**0.23**	**-0.98 – -0.06**	**-2.21***
	**R^2=0.03, F(1, 393)=11.44, p=.001**				**R^2=0.01, F(1,393)=4.74, p=.03**			
Model 2	**0.40**	**0.15**	**0.10 - 0.70**	**2.61** **	**-0.39**	**0.19**	**-0.76 - -0.03**	**-2.10***
	**R^2=0.43, F(18,376)=15.97, p<.001**				**R^2=0.39, F(18,376)=9.58, p<.001**			
**Months 3-6 post birth**								
	B	SE	95% CI	t	B	SE	95% CI	t
Model 1	0.32	0.22	-0.11 – 0.76	1.47	-0.25	0.27	-0.78 – 0.28	-0.93
	R^2=0.01, F(1,305)=2.17, p=.14				R^2=0.003, F(1,301)=0.86, p=.35			
Model 2	0.20	0.16	-0.12 – 0.52	1.23	-0.18	0.21	-0.59 – 0.23	-0.86
	R^2=0.44, F(18,284)=11.22, p<.001				R^2=0.37, F(18,280)=7.19, p<.001			

*Model 1: unadjusted; Model 2: adjusted for baseline wellbeing/depression, mother's age, maternal education status, household income and number of previous children, as well as how many weeks the baby was post-birth, and the mother's marital status at T2, whether she was working at T1 or T2 and whether her partner was working at T2 and previous histories of both anxiety and depression. *p<.05, **p<.01, ***p<.001*

between listening and baseline depression (B=-0.09, SE=0.04, 95%CI -0.17 to -0.01), with a two-way contour graph showing that listening to music particularly seemed to support those who were showing symptoms of PND (evidenced with a score of 10+ at baseline) (see Supplementary Figure 1B).

Finally, in order to try and ascertain whether listening to music led to changes in mental health or whether mental health led to changes in listening habits, we ran additional analyses reversing the variable order. While this does not confirm potential causal mechanisms, it can give an indication as to whether mental health can predict listening behaviours and therefore support hypotheses about temporal precedence. There was no evidence that levels of wellbeing during pregnancy were associated with the likelihood of listening to music either 3 or 6 months post-birth. However, there was some indication that depression symptoms in the final trimester of pregnancy were associated with listening habits 3 months post-birth (β=-0.03, SE=0.01, 95%CI -0.05 to -0.01, p=.003).

Discussion

This study explored associations between listening to music in the final trimester of pregnancy and mental health and wellbeing in mothers post-birth. Listening was found to be associated with higher

For peer review only - http://bmjopen.bmj.com/site/about/guidelines.xhtml

levels of wellbeing and reduced symptoms of PND in the first 3 months post-birth, even when adjusting for baseline mental health and potential confounding variables. These results appear to be particularly found amongst women with lower levels of wellbeing at baseline. These findings echo the few existing studies in showing that listening to music is associated with better mental health in the perinatal period (30,31). However, to the authors' knowledge, this is the first study to show that listening to music during pregnancy is longitudinally associated with better mental health post-birth.

Across both symptoms of PND and wellbeing, however, associations were only found for the first 3 months post-birth, and had disappeared by the second quartile post-birth. The hypnosis study previously described also found results within the first 3 months post-partum (weeks 2 and 10) but did not measure beyond this, so there is little data available against which to benchmark these findings (16). Nevertheless, the immediate period post-birth has been highlighted as being of particular challenge for new mothers, with the transition into assuming maternal tasks and adjusting to the new role lasting until around the third month postpartum (13,34). It is possible, therefore, that any effects of music listening during the prenatal period are of most value during this transition period, but become less significant once mothers and their babies become more settled.

A key question is how listening to music is associated with better mental health and wellbeing in the postnatal period. There are a number of potential explanations. First, studies involving psychological tests, neuroimaging, biomarker analyses and ethnographic observations have shown that listening to music can have marked effects on stress and anxiety (25). Specifically in relation to pregnant women, listening to music for just 30 minutes can reduce cortisol levels and anxiety (29). Wider studies involving listening to music have shown it to be particularly effective at reducing psychological and physiological responses to stress, especially when people deliberately listen to music in order to help them relax (35,36). This effect of music on stress has in turn been linked specifically through to theories around wellbeing (25,37), with a wide literature linking stress and anxiety with both mental health and wellbeing (38,39). It is proposed that high levels of anxiety might hinder women's adaptation to motherhood in the initial postpartum period, with negative effects on wellbeing (40). Consequently, it is possible that the relaxing effects of listening to music during the pregnancy period helps to act as a buffer for feelings of stress and anxiety, thereby supporting mothers in maintaining their adaptation and leading to enhanced wellbeing.

Another potential explanation relates to the effects of music on mood. Mood regulation has been identified as one of the prime reasons why people listen to music, with models of mood regulation by music highlighting its effects on mood-related subjective experience (including the intensity and clarity of moods), physiological responses (such as energy levels and movement) and behaviours (such as their ability to express emotions) (41). Music listening has been found to modulate

For peer review only - http://bmjopen.bmj.com/site/about/guidelines.xhtml

depression and wellbeing (42,43). Early low mood during the prenatal period is directly associated with lower wellbeing and postnatal depression post-birth (44), leading to propositions that interventions that deliberately attempt to cultivate positive emotions, such as relaxation therapies and interventions focused on finding positive meaning, could directly optimise health and wellbeing in this population. Consequently, it is possible that another route by which listening to music in the third trimester of pregnancy is associated with improvements in mental health and wellbeing is via enhancing mood.

Finally, a third explanation is that listening to music in itself did not have an effect post-birth but did enhance coping skills in women while they were still pregnant, which in turn led to higher wellbeing post-birth. Music listening has been linked with both problem-oriented coping and emotion-oriented coping, specifically with results showing that problem-oriented coping by music listening in women is linked to lower depression levels (45). Life transitions (such as the perinatal period) depend on both health and wellbeing and also on appraisal and coping responses. In the hypnosis study previously mentioned, the authors proposed that the intervention during pregnancy helped mothers to maintain and enhance their wellbeing whilst pregnant, which in turn influenced their appraisal of the perinatal transition period and supported their coping responses (16). It is possible that a similar process took place through listening to music, with listening to music supporting coping in the prenatal period, which encouraged mothers' own coping skills, which in turn led to better coping during and post-birth and consequent higher wellbeing. Indeed, even just in relation to the birth, a number of birth preparation courses focus on relaxation and mood optimisation, which have been shown to lead to less negative affect and better coping during labour and delivery (46). Given that a significant predictor of PND is the birth experience, enhanced coping prior to the birth, perhaps through music listening, could be an important factor in postnatal wellbeing (47).

This study has a number of limitations. First, the study followed a cohort of women rather than being interventional, so it is not possible to confirm causality. However, the study had a longitudinal design, there was no evidence of reverse causality in relation to wellbeing, there are plausible proposed mechanistic explanations, and there is a strong body of previous literature causally linking music and mental health in other populations. So this study provides promising preliminary evidence that remains to be tested in a future experimental design. A second limitation is that the population in this study was not nationally representative. Nevertheless, there was a clear spread of participants from varying socio-economic backgrounds as well as variations in the levels of exposure and outcome variables. So the dataset provides interesting and suitable preliminary data on the longitudinal associations between music listening and mental health. Third, this study explored the impact of all music listening, not specifying particular genres. Previous research has suggested that certain genres of music (or more specifically compositional aspects of music such as its valence and arousal levels)

For peer review only - http://bmjopen.bmj.com/site/about/guidelines.xhtml

can lead to different responses, such as variations in relaxation or mood (48). However, most of these genre-specific effects have been found in tightly-controlled lab-based studies, and literature from real-world interaction with music has suggested that musical preference might be more important in determining the effects of music (49). This study followed these real-world studies in recording what genres people did listen to but measuring the quantity of listening based on preference rather than genre. Future studies could explore the impact of different genres on mental health in the perinatal period.

In conclusion, this study provides the first preliminary evidence that listening to music during the third trimester of pregnancy could be protective against symptoms of PND and low wellbeing in the first three months post-birth. Music listening is an attractive intervention in that it is readily available to people from all echelons of society regardless of socioeconomic status, educational attainment or cultural background. It can be carried out in a range of contexts so is not restricted to particular places or times. It is also inexpensive: indeed the majority of women in the Western countries have access to recorded music already. Finally, there are no obvious side effects from listening to music. Consequently, listening to music could be recommended as a way of supporting pregnant women; in particular those who demonstrate low wellbeing or symptoms of PND.

Authors' contributions

DF and RP designed the study and collected data. DF ran the analyses and drafted the paper. Both authors critically revised the manuscript and approved it for submission.

Data sharing statement

The data in this study are available from the authors upon request.

Patient public involvement

This study was developed as part of a wider grant that involved mothers, psychiatrists and health workers in the design of the research questions, the choice of measures and the recruitment for the study. We also involved these groups in the dissemination of the results.

For peer review only

References

1. Khalifeh H, Brauer R, Toulmin H, Howard LM. Perinatal mental health: What every neonatologist should know. Early Hum Dev. 2015 Nov 1;91(11):649–53.
2. Morrell C, Warner R, Slade P, Dixon S, Walters S, Paley G, et al. Psychological interventions for postnatal depression: cluster randomised trial and economic evaluation. The PoNDER trial. Health Technol Assess [Internet]. 2009 Jun [cited 2016 Jul 26];13(30). Available from: http://www.journalslibrary.nihr.ac.uk/hta/volume-13/issue-30
3. Cooper PJ, Murray L. Postnatal depression. BMJ. 1998 Jun 20;316(7148):1884–6.
4. Junge C, Garthus-Niegel S, Slinning K, Polte C, Simonsen TB, Eberhard-Gran M. The Impact of Perinatal Depression on Children's Social-Emotional Development: A Longitudinal Study. Matern Child Health J. 2017 Mar 1;21(3):607–15.
5. Green JM, Kafetsios K. Positive experiences of early motherhood: Predictive variables from a longitudinal study. J Reprod Infant Psychol. 1997 May 1;15(2):141–57.
6. Keyes CL. Mental Illness and/or Mental Health? Investigating Axioms of the Complete State Model of Health. J Consult Clin Psychol. 2005;73(3):539–48.
7. Boyce PM, Johnstone SJ, Hickey AR, Morris-Yates AD, Harris MG, Strachan T. Functioning and well-being at 24 weeks postpartum of women with postnatal depression. Arch Womens Ment Health. 2000 Dec 11;3(3):91–7.
8. Jomeen J. The importance of assessing psychological status during pregnancy, childbirth and the postnatal period as a multidimensional construct: A literature review. Clin Eff Nurs. 2004 Sep;8(3–4):143–55.
9. Allan C, Carrick-Sen D, Martin CR. What is perinatal well-being? A concept analysis and review of the literature: J Reprod Infant Psychol [Internet]. 2013 [cited 2017 Jun 8];31(4). Available from: http://www.tandfonline.com/doi/abs/10.1080/02646838.2013.791920
10. Beck CT. Predictors of postpartum depression: an update. Nurs Res. 2001 Oct;50(5):275–85.
11. Milgrom J, Gemmill AW, Bilszta JL, Hayes B, Barnett B, Brooks J, et al. Antenatal risk factors for postnatal depression: a large prospective study. J Affect Disord. 2008 May;108(1–2):147–57.
12. Robertson E, Grace S, Wallington T, Stewart DE. Antenatal risk factors for postpartum depression: a synthesis of recent literature. Gen Hosp Psychiatry. 2004 Jul;26(4):289–95.
13. Leifer M. Psychological effects of motherhood : a study of first pregnancy / Myra Leifer. New York: Praeger; 1980.
14. Elliott SA, Leverton TJ, Sanjack M, Turner H, Cowmeadow P, Hopkins J, et al. Promoting mental health after childbirth: a controlled trial of primary prevention of postnatal depression. Br J Clin Psychol. 2000 Sep;39 (Pt 3):223–41.
15. Spinelli MG. Prevention of postpartum mood disorders. In: Postpartum mood disorders. Arlington, VA, US: American Psychiatric Association; 1999. p. 217–35.
16. Guse T, Wissing M, Hartman W. The effect of a prenatal hypnotherapeutic programme on postnatal maternal psychological well□being. J Reprod Infant Psychol. 2006 May;24(2):163–77.
17. Chan MF, Wong ZY, Thayala NV. The effectiveness of music listening in reducing depressive symptoms in adults: A systematic review. Complement Ther Med. 2011 Dec 1;19(6):332–48.
18. Cassidy JW, Standley JM. The Effect of Music Listening on Physiological Responses of Premature Infants in the NICU. J Music Ther. 1995 Dec 21;32(4):208–27.
19. Pelletier CL. The effect of music on decreasing arousal due to stress: a meta-analysis. J Music Ther. 2004 Fall;41(3):192–214.
20. Siedliecki SL, Good M. Effect of music on power, pain, depression and disability. J Adv Nurs. 2006 Jun 1;54(5):553–62.

For peer review only - http://bmjopen.bmj.com/site/about/guidelines.xhtml

21. Hsu W-C, Lai H-L. Effects of music on major depression in psychiatric inpatients. Arch Psychiatr Nurs. 2004 Oct 1;18(5):193–9.
22. Chan MF, Chan EA, Mok E, Kwan Tse FY. Effect of music on depression levels and physiological responses in community-based older adults. Int J Ment Health Nurs. 2009 Aug 1;18(4):285–94.
23. Laukka P. Uses of music and psychological well-being among the elderly. J Happiness Stud. 2007 Jun 1;8(2):215.
24. Haake AB. Individual music listening in workplace settings An exploratory survey of offices in the UK. Music Sci. 2011 Mar 1;15(1):107–29.
25. Vastfjall D, Juslin PN, Hartig T. Music, subjective wellbeing, and health: the role of everyday emotions. In: MacDonald R, Kreutz G, Mitchell L, editors. Music, Health, and Wellbeing. Oxford University Press; 2013.
26. McCaffrey R. Music Listening: Its Effects in Creating a Healing Environment. J Psychosoc Nurs Ment Health Serv. 2008 Oct 1;46(10):39–44.
27. Batt-Rawden KB, DeNora T, Ruud E. Music Listening and Empowerment in Health Promotion: A Study of the Role and Significance of Music in Everyday Life of the Long-term Ill. Nord J Music Ther. 2005 Jul 1;14(2):120–36.
28. Skånland MS. Everyday music listening and affect regulation: The role of MP3 players. Int J Qual Stud Health Well-Being [Internet]. 2013 [cited 2017 Jun 14];8. Available from: /pmcc/articles/PMC3740499/?report=abstract
29. Ventura T, Gomes MC, Carreira T. Cortisol and anxiety response to a relaxing intervention on pregnant women awaiting amniocentesis. Psychoneuroendocrinology. 2012 Jan;37(1):148–56.
30. Nwebube C, Glover V, Stewart L. Prenatal listening to songs composed for pregnancy and symptoms of anxiety and depression: a pilot study. BMC Complement Altern Med. 2017 May 8;17(1):256.
31. Fancourt D, Perkins R. Associations between singing to babies and symptoms of postnatal depression, wellbeing, self-esteem and mother-infant bond. Public Health. 2017 Apr 1;145:149–52.
32. Cox JL, Holden JM, Sagovsky R. Detection of postnatal depression. Development of the 10-item Edinburgh Postnatal Depression Scale. Br J Psychiatry. 1987 Jun 1;150(6):782–6.
33. Stewart-Brown S, Tennant A, Tennant R, Platt S, Parkinson J, Weich S. Internal construct validity of the Warwick-Edinburgh Mental Well-being Scale (WEMWBS): a Rasch analysis using data from the Scottish Health Education Population Survey. Health Qual Life Outcomes. 2009;7:15.
34. Pridham KF, Chang AS. Transition to being the mother of a new infant in the first 3 months: maternal problem solving and self-appraisals. J Adv Nurs. 1992 Feb;17(2):204–16.
35. Krout RE. The effects of single-session music therapy interventions on the observed and self-reported levels of pain control, physical comfort, and relaxation of hospice patients. Am J Hosp Palliat Med. 2001 Dec;18(6):383–90.
36. Linnemann A, Strahler J, Nater UM. The stress-reducing effect of music listening varies depending on the social context. Psychoneuroendocrinology. 2016 Oct 1;72:97–105.
37. Krout RE. Music listening to facilitate relaxation and promote wellness: Integrated aspects of our neurophysiological responses to music. Arts Psychother. 2007;34(2):134–41.
38. Iwata M, Ota KT, Duman RS. The inflammasome: pathways linking psychological stress, depression, and systemic illnesses. Brain Behav Immun. 2013 Jul;31:105–14.
39. Vedhara K, Miles J, Bennett P, Plummer S, Tallon D, Brooks E, et al. An investigation into the relationship between salivary cortisol, stress, anxiety and depression. Biol Psychol. 2003 Feb;62(2):89–96.
40. Pond E, Kemp V. A comparison between adolescent and adult women on prenatal anxiety and self-confidence. Matern Child Nurs J. 1992;20(1):11–20.

For peer review only - http://bmjopen.bmj.com/site/about/guidelines.xhtml

41. Saarikallio S, Erkkilä J. The role of music in adolescents' mood regulation. Psychol Music. 2007 Jan 1;35(1):88–109.
42. Chin T, Rickard NS. Emotion regulation strategy mediates both positive and negative relationships between music uses and well-being. Psychol Music. 2014 Sep 1;42(5):692–713.
43. Lai Y-M. Effects of Music Listening on Depressed Women in Taiwan. Issues Ment Health Nurs. 1999 Jan 1;20(3):229–46.
44. Fleming A, N. Ruble D, L. Flett G, L. Shaul D. Postpartum Adjustment in First-Time Mothers: Relations Between Mood, Maternal Attitudes, and Mother-Infant Interactions. Dev Psychol. 1988 Jan 1;24:71–81.
45. Miranda D, Claes M. Music listening, coping, peer affiliation and depression in adolescence. Psychol Music. 2009 Apr 1;37(2):215–33.
46. Duncan S, J. Markman H. Intervention programs for the transition to parenthood: Current status from a prevention perspective. 2017 Oct 26;
47. Green JM. Expectations and experiences of pain in labor: findings from a large prospective study. Birth Berkeley Calif. 1993 Jun;20(2):65–72.
48. Labbe E, Schmidt N, Babin J, Pharr M. Coping with stress: the effectiveness of different types of music. Appl Psychophysiol Biofeedback. 2007 Dec;32(3–4):163–8.
49. Fancourt D, Ockelford A, Belai A. The psychoneuroimmunological effects of music: A systematic review and a new model. Brain Behav Immun. 2014 Feb;36:15–26.

For peer review only

For peer review only - http://bmjopen.bmj.com/site/about/guidelines.xhtml

Supplementary Figure 1: Contour graphs showing the interaction between baseline mental health and music listening habits on mental health post-birth

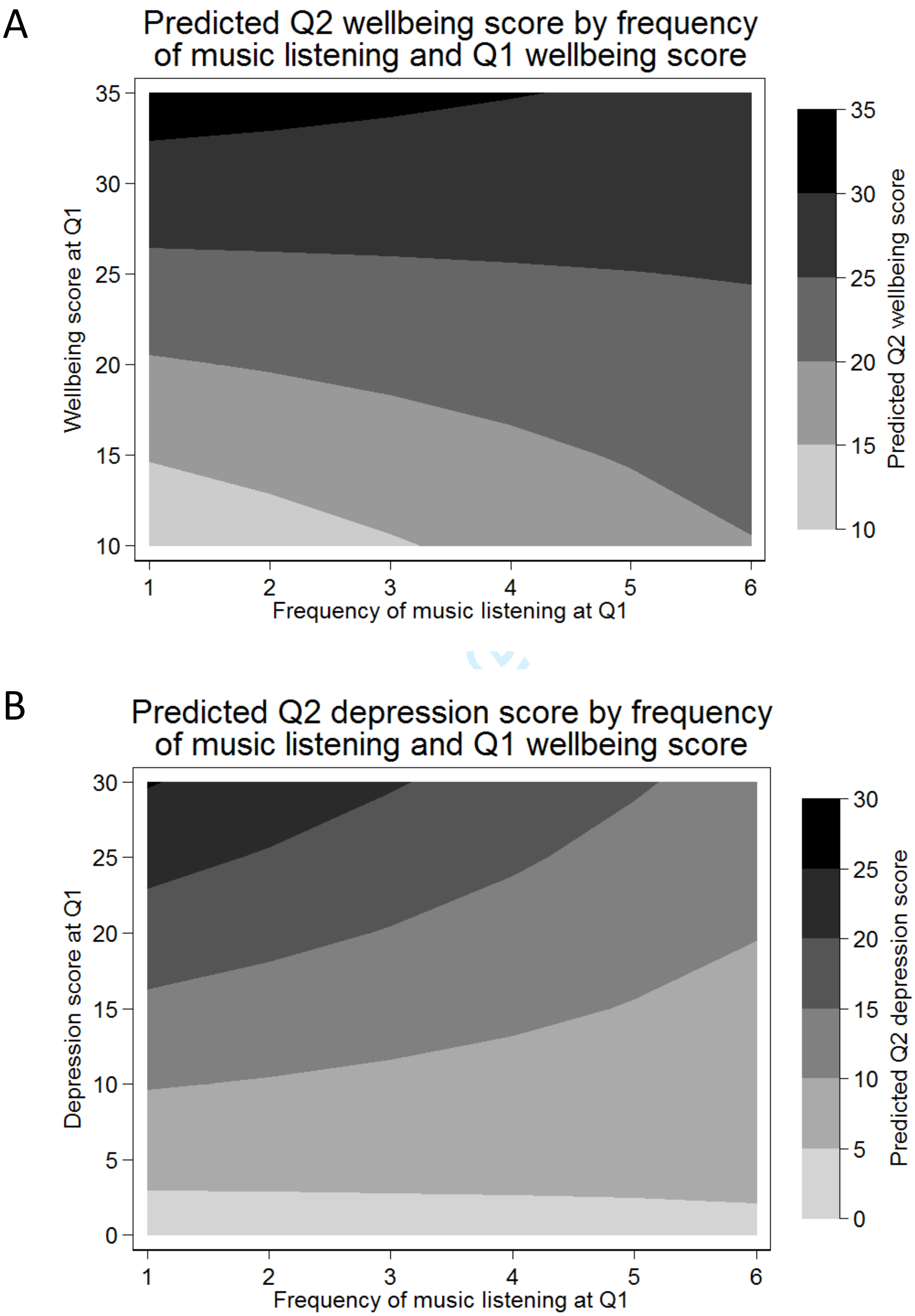

Notes. **Frequency of music listening at Q1:** 1=rarely; 2=a couple of times a week; 3=every day <1hr; 4=every day 1-2hrs; 5=every day 3-5hrs; 6=every day 5+hrs

STROBE Statement—Checklist of items that should be included in reports of ***cohort studies***

	Item No	Recommendation	Page
**Title and abstract**	1	(*a*) Indicate the study's design with a commonly used term in the title or the abstract	1
		(*b*) Provide in the abstract an informative and balanced summary of what was done and what was found	2
**Introduction**			
Background/rationale	2	Explain the scientific background and rationale for the investigation being reported	4-5
Objectives	3	State specific objectives, including any prespecified hypotheses	5
**Methods**			
Study design	4	Present key elements of study design early in the paper	6
Setting	5	Describe the setting, locations, and relevant dates, including periods of recruitment, exposure, follow-up, and data collection	6
Participants	6	(*a*) Give the eligibility criteria, and the sources and methods of selection of participants. Describe methods of follow-up	6
		(*b*) For matched studies, give matching criteria and number of exposed and unexposed	
Variables	7	Clearly define all outcomes, exposures, predictors, potential confounders, and effect modifiers. Give diagnostic criteria, if applicable	6-7
Data sources/ measurement	8*	For each variable of interest, give sources of data and details of methods of assessment (measurement). Describe comparability of assessment methods if there is more than one group	6-7
Bias	9	Describe any efforts to address potential sources of bias	7-8
Study size	10	Explain how the study size was arrived at	
Quantitative variables	11	Explain how quantitative variables were handled in the analyses. If applicable, describe which groupings were chosen and why	6-7
Statistical methods	12	(*a*) Describe all statistical methods, including those used to control for confounding	7
		(*b*) Describe any methods used to examine subgroups and interactions	7-8
		(*c*) Explain how missing data were addressed	8
		(*d*) If applicable, explain how loss to follow-up was addressed	8
		(*e*) Describe any sensitivity analyses	7-8
**Results**			
Participants	13*	(a) Report numbers of individuals at each stage of study—eg numbers potentially eligible, examined for eligibility, confirmed eligible, included in the study, completing follow-up, and analysed	6
		(b) Give reasons for non-participation at each stage	
		(c) Consider use of a flow diagram	
Descriptive data	14*	(a) Give characteristics of study participants (eg demographic, clinical, social) and information on exposures and potential confounders	6
		(b) Indicate number of participants with missing data for each variable of interest	N/A
		(c) Summarise follow-up time (eg, average and total amount)	6
Outcome data	15*	Report numbers of outcome events or summary measures over time	8-9
Main results	16	(*a*) Give unadjusted estimates and, if applicable, confounder-adjusted estimates and their precision (eg, 95% confidence interval). Make clear which	8-9

For peer review only

		confounders were adjusted for and why they were included	
		(*b*) Report category boundaries when continuous variables were categorized	
		(*c*) If relevant, consider translating estimates of relative risk into absolute risk for a meaningful time period	
Other analyses	17	Report other analyses done—eg analyses of subgroups and interactions, and sensitivity analyses	9-10
**Discussion**			
Key results	18	Summarise key results with reference to study objectives	10
Limitations	19	Discuss limitations of the study, taking into account sources of potential bias or imprecision. Discuss both direction and magnitude of any potential bias	12
Interpretation	20	Give a cautious overall interpretation of results considering objectives, limitations, multiplicity of analyses, results from similar studies, and other relevant evidence	12
Generalisability	21	Discuss the generalisability (external validity) of the study results	12
**Other information**			
Funding	22	Give the source of funding and the role of the funders for the present study and, if applicable, for the original study on which the present article is based	3

*Give information separately for exposed and unexposed groups.

Note: An Explanation and Elaboration article discusses each checklist item and gives methodological background and published examples of transparent reporting. The STROBE checklist is best used in conjunction with this article (freely available on the Web sites of PLoS Medicine at http://www.plosmedicine.org/, Annals of Internal Medicine at http://www.annals.org/, and Epidemiology at http://www.epidem.com/). Information on the STROBE Initiative is available at http://www.strobe-statement.org.

For peer review only

For peer review only - http://bmjopen.bmj.com/site/about/guidelines.xhtml

BMJ Open

Could listening to music during pregnancy be protective against postnatal depression and poor wellbeing post-birth? Longitudinal associations from a preliminary prospective cohort study.

Journal:	*BMJ Open*
Manuscript ID	bmjopen-2017-021251.R2
Article Type:	Research
Date Submitted by the Author:	24-May-2018
Complete List of Authors:	Fancourt, Daisy; University College London Research Department of Epidemiology and Public Health, Department of Behavioural Science and Health<br>Perkins, Rosie; Royal College of Music, Centre for Performance Science; Imperial College London, Faculty of Medicine
**Primary Subject Heading**:	Mental health
Secondary Subject Heading:	Obstetrics and gynaecology
Keywords:	MENTAL HEALTH, Depression & mood disorders < PSYCHIATRY, Maternal medicine < OBSTETRICS

SCHOLARONE™
Manuscripts

For peer review only

Could listening to music during pregnancy be protective against postnatal depression and poor wellbeing post-birth? Longitudinal associations from a preliminary prospective cohort study.

Daisy Fancourt PhD [a,b] and Rosie Perkins PhD [b,c]*

[a] Department of Behavioural Science and Health, University College London, London WC1E 7HB

[b] Faculty of Medicine, Imperial College London, London SW7 2AZ

[c] Centre for Performance Science, Royal College of Music, London SW7 2BS

* Corresponding author: Dr Daisy Fancourt, 1-19 Torrington Place, London, WC1E 7HB, d.fancourt@ucl.ac.uk

For peer review only

Abstract

Objectives: This study explored whether listening to music during pregnancy is longitudinally associated with lower symptoms of postnatal depression and higher wellbeing in mothers post-birth.

Design: Prospective cohort study.

Participants: We analysed data from 395 new mothers aged over 18 who provided data in the third trimester of pregnancy and 3 and 6 months later (0-3 and 4-6 months post birth).

Primary and secondary outcome measures: Postnatal depression was measured using the Edinburgh Postnatal Depression Scale (EPDS), and wellbeing was measured using the Short Warwick-Edinburgh Mental Wellbeing Scale (SWEMWBS). Our exposure was listening to music and was categorised as 'rarely; a couple of times a week; every day <1hr; every day 1-2hrs; every day 3-5hrs; every day 5+hrs'. Multivariable linear regression analyses were carried out to explore the effects of listening to music during pregnancy on depression and wellbeing post birth, adjusted for baseline mental health and potential confounding variables.

Results: Listening during pregnancy is associated with higher levels of wellbeing (β=0.40, SE=0.15, 95%CI 0.10 to 0.70) and reduced symptoms of postnatal depression (β=-0.39, SE=0.19, 95%CI -0.76 to -0.03) in the first 3 months post-birth. However, effects disappear by 4-6 months post-birth. These results appear to be particularly found amongst women with lower levels of wellbeing and high levels of depression at baseline.

Conclusions: Listening to music could be recommended as a way of supporting mental health and wellbeing in pregnant women; in particular those who demonstrate low wellbeing or symptoms of postnatal depression.

Keywords

Postnatal depression, wellbeing, mental health, music, perinatal

Strengths and limitations of this study

- This preliminary prospective cohort study tracked a sample of women across the perinatal period providing data at 12 week intervals.
- The data include a rich set of variables on music listening behaviours amongst participants.

For peer review only

- We adjusted for all identified confounding variables in our analyses and ran sensitivity analyses to test our assumptions.
- The data are not nationally representative, although there is a clear spread of participants from varying socio-economic backgrounds as well as variations in the levels of exposure and outcome variables.
- As this is a cohort study and not interventional, it is not possible to confirm causality.

Funding statement

The study was funded by Arts Council England Research Grants Fund, grant number 29230014 (Lottery). D.F. is supported by the Wellcome Trust [205407/Z/16/Z]. The study was approved by the NHS REC (15/SS/016). The study team acknowledge the support of the National Institute of Health Research Clinical Research Network (NIHR CRN). The authors would like to thank the hospitals involved as Participant Identification Centres as well as Miss Sunita Sharma, Prof Aaron Williamon and Sarah Yorke for their support with the study.

Competing interests statement

Both authors declare no conflict of interest.

For peer review only

Introduction

Perinatal mental health problems affect around 20% of women at some point during the perinatal period (1). In terms of conditions characterised by negative symptomology, postnatal depression (PND) is one of the most common problems, and is a debilitating condition with symptoms including fatigue, irritability, insomnia and anhedonia; symptoms which in 25% of affected women last for at least one year (2). Over the last two decades, there has been significant research into the effects of PND on mother and infant as well as attention paid to how it can be prevented or managed (3,4). However, in terms of conditions relating more to the absence of positive symptomology, such as low hedonic or eudemonic wellbeing, there has been much less research. The few studies that do exist have found that negative mood, as indicated by the Edinburgh Postnatal Depression Scale (EPDS), has a correlation of just -0.46 with positive experiences of motherhood, as indicated by a principal component analysis of six experiences of motherhood (POSMO) (5). This suggests that, as in the wider population, depression and wellbeing are separate constructs in the context of perinatal mental health and a positive perinatal experience is more than simply the inversion of negative mood (6). Building on this, women even without PND have been found to demonstrate impairments in emotional problems and vitality, suggesting that even in the absence of depression mothers can have impaired wellbeing (7). In light of this, a review has argued that psychological wellbeing defined as a multidimensional construct should be an integral part of maternity care (8), and a more recent construct analysis has highlighted the importance not just of identifying PND but also of identifying women with sub-optimal perinatal wellbeing and supporting them to achieve positive psychological functioning (9).

In seeking to support the perinatal mental health of women, the pregnancy period has been highlighted as critical. Prenatal mental health has repeatedly been shown to be one of the largest predictors of postnatal depression (10–12) and wellbeing (9). In particular, the third trimester of pregnancy has been identified as an important transition period involving adaptation to emotional and physical changes, leading to feelings of wellbeing often less pronounced than in the previous trimesters (13). Early detection of symptoms of depression and low wellbeing during pregnancy and prompt intervention is therefore important in reducing adverse consequences.

In light of this, there are a number of interventions that have been developed to try and support mental health in the prenatal period as a way of reducing postnatal mental health problems, in particular focusing on the third trimester as a point of intervention. There have been findings that support the application of cognitive-behavioural and interpersonal psychotherapy, suggesting that depression following childbirth could be prevented by brief interventions in the prenatal period (14,15). In exploring other interventions, prenatal hypnotherapy has been found to significantly reduce PND and improve psychological wellbeing at two weeks and ten weeks postpartum (16). And a psychosocial

For peer review only - http://bmjopen.bmj.com/site/about/guidelines.xhtml

intervention involving group meetings to discuss aspects of parenthood in the final trimester of pregnancy and first 6 months postpartum has been found to reduce PND amongst first-time mothers (14). However, as many mothers continue to work full-time until shortly before their due dates, in-person interventions may not be feasible for all mothers and are of course limited by what is available in different geographical areas. As a result, there is a need to identify other home-based interventions that could provide similar mental health support.

Over the past two decades, there has been increasing research showing the effects of listening to music on mental health. A number of reviews have demonstrated the effects of regular music listening including in enhancing mental health in the general population (17), reducing distress in premature infants (18), and reducing stress in adults (19). Specifically in relation to depression, listening to music has been shown to reduce depression amongst adults with chronic pain (20), psychiatric inpatients (21), and older adults (17,22). In relation to wellbeing, music listening has been shown to be associated with better wellbeing not just in controlled interventions but also as a result of ordinary day-to-day listening. A Swedish study involving 500 older adults found associations between music listening and wellbeing, even when controlling for potential confounding variables (23). Studies tracking daily activities have linked music listening with enhanced wellbeing both in the workplace and in the wider context of people's lives (24,25). Further, music has also been shown to contribute to creating supportive healthy environments, connecting individuals with their emotions and promoting wellbeing (26). Finally, theoretical studies have highlighted the role of music listening in enhancing affect, wellness and resources for recovery and quality of life (27,28). Consequently, both directed music listening interventions and routine day-to-day music listening can affect levels of depression and wellbeing in a range of different populations.

Specifically in relation to the perinatal period, a few studies have suggested that music listening may be supportive for mental health. Listening to music for just 30 minutes has been found to reduce cortisol levels and anxiety in pregnant women, leading to recommendations that pregnant women might benefit from regular listening to music as a practice of relaxation (although the effects of regular listening were not tested) (29). A recent study found that women who listened to recorded music for 20 minutes a day for 12 weeks during their pregnancy had significant improvements in anxiety and depression (30). However, the study did not track outcomes postnatally and involved a small sample of women. And a further study has found cross-sectional associations between listening to music and depression and wellbeing amongst new mothers, with more frequent listening associated with better mental health (31). However, this study did not look longitudinally nor involve pregnant women.

For peer review only - http://bmjopen.bmj.com/site/about/guidelines.xhtml

Therefore, to date, despite promising results suggesting that listening to music can modulate mental health and wellbeing during the perinatal period, no studies have looked specifically at the impact of listening to music during pregnancy on depression and wellbeing post-birth. In order to address this research gap, this study tracked a cohort of mothers across the perinatal period in order to ascertain whether there was a relationship between music listening during pregnancy and postnatal mental health.

Methods

Participants and procedure

This study used data collected as part of a larger study exploring the impact of creative interventions on perinatal mental health. Women living in England in the last trimester of pregnancy (28 weeks or more) and the first nine months post birth (up to 40 weeks) were recruited from hospitals, General Practices, mother and baby charities and through social media in England across October 2015 to March 2016, and completed an anonymous cross-sectional online questionnaire. Women in the final trimester of pregnancy (28 weeks or more) were then invited to continue their participation in a longitudinal study. This involved providing another wave of data 3 months, 6 months and 9 months following the first date of data collection (which equated to providing baseline data T1 during pregnancy, T2 data in the first 3 months post-birth, T3 data in months 4-6 post-birth and T4 data in months 7-9 post-birth). From an initial sample of 550 mothers who consented to be involved in this longitudinal study, a total of 458 mothers provided T2 data (83%), 417 provided T3 data (75.8%) and 392 (71.3%) provided T4 data. The study received ethical approval from the UK NHS Research Ethics Service and all participants gave informed consent prior to involvement in the research.

For this study, and in light of the literature review presented above, we hypothesised that listening during pregnancy would support wellbeing and reduce symptoms of PND in the first trimester post-birth. We therefore focused on women in the longitudinal study who had provided complete data on the variables we selected for analyses at both T1 and T2: 395 women. However, we also ran some exploratory follow-up analyses with women who had also provided complete data at T3 (n=299) in order to explore if effects were maintained.

Patient public involvement

This study was developed as part of a wider grant that involved mothers, psychiatrists and health workers in the design of the research questions, the choice of measures and the recruitment for the study. We also involved these groups in the dissemination of the results.

Measures

For peer review only - http://bmjopen.bmj.com/site/about/guidelines.xhtml

Symptoms of PND were measured using the Edinburgh Postnatal Depression Scale (EPDS), a 10-item scale used extensively both with pregnant women and new mothers, scored from 0-30 with 10+ indicative of possible symptoms of depression and higher scores of 13+ indicating more severe depression (32).

Wellbeing was measured using the Short Warwick-Edinburgh Mental Wellbeing Scale (SWEMWBS), a scale that encompasses both hedonic and eudemonic wellbeing comprising 7 items scored from 7 to 35 with higher scores representing higher levels of wellbeing. The raw scores were logit-transformed prior to analysis (33). The New Economics Foundation (NEF) suggests five levels of wellbeing based on quintile analyses of data in the UK Understanding Society Survey, 2009: poor (<22), below average (22-24), average (25-26), good (27-28) and excellent (29-35).

In addition, demographic variables assessed the women's number of weeks pregnant/post-birth, number of other children (0, 1, 2, 3 and 4+), household income (<£16,000, £16,000-£30,000, £31,000-£60,000, £61,000-£90,000, >£90,000), educational attainment (school to 16, sixth form/college, undergraduate degree, postgraduate degree), marital status (married vs not married), employment status (working vs not working), partner's employment status (working vs not working) and whether the woman had previously been diagnosed with either anxiety or depression.

Listening to music was categorised as 'rarely; a couple of times a week; every day <1hr; every day 1-2hrs; every day 3-5hrs; every day 5+hrs'. While these analyses focused on quantity of music listening as a predictor, we also recorded genre of music listened to.

<u>Statistics</u>

Data were analysed using Stata v14. Multivariable linear regression models were used to explore the effects of listening to music on wellbeing and PND. Frequency of listening to music had a normal distribution so was treated as a 6-point linear variable, with higher score indicating more frequent listening. For wellbeing and PND, we used raw scores. Model 1 was unadjusted, while Model 2 adjusted for baseline wellbeing/depression, mother's age, maternal education status, household income and number of previous children, as well as how many weeks the baby was post-birth, and the mother's marital status at T2, whether she was working at T1 or T2, whether her partner was working at T2, and previous histories of both anxiety and depression.

All models displayed linearity as assessed by augmented partial residual plots with lowess smoothing; multicollinearity as assessed by checking variance inflation factors; normality as assessed using kernel density plots, standardised normal probability (P-P) plots and Q-Q plots; and there was no evidence of outliers or undue influence as assessed using added variable plots regressing each variable

For peer review only

against all others, through stem and leaf plots, and through assessing covariance ratios, Cook's distance and leverage. The wellbeing regression models demonstrated homoscedasticity as assessed by plotting the residuals versus fitted (predicted) values and using the Breusch-Pagan test for heteroskedasticity. However, the depression regression models showed signs of heteroskedasticity, so robust standard errors were calculated.

Planned sensitivity analyses were then conducted in order to ascertain whether baseline mental health during pregnancy was a moderator of the association between listening to music and mental health post birth. For this, we included an interaction term of Q1 mental health and Q1 listening habits in our regression models and then plotted two-way contour graphs to visualise the interaction (see Supplementary Figure 1).

Although there were no significant demographic differences between those who did and did not provide data at Q3, we wanted to take account of potential minor demographic differences between those who provided data at Q3 and those who failed to. So the propensity score for non-response was calculated using the indicators listed in Model 2 above (none of which significantly predicted missingness) and inverse probability weighting was applied to the T3 regression models. We confirmed goodness of fit using the Hosmer-Lemeshow test. Weighted analyses did not differ from unweighted analyses.

Results

Demographics. At T1, women had an average age of 31.9 years (SD=4.9, range 18-47) and an average of 32.9 weeks pregnant (SD=4.1, range 28-42). Further demographics are provided in Table 1.

Table 1: Demographic information on participants

	N=395
Maternal age, μ (SD)	31.9 (4.9)
Infant age, μ (SD)	32.9 (4.1)
Marital status, %	
Married	69.3
Cohabiting	25.9
In a relationship but living separately	3.8
Single	1
Partner working, %	97.0
Educational attainment, %	
Education to 16)	13.2
Education to 18	16.5
Undergraduate degree/qualification	41.3
Postgraduate degree/qualification	29.1
Household income, %	

For peer review only - http://bmjopen.bmj.com/site/about/guidelines.xhtml

< £16,000	6.6
£16,000-£30,000	11.1
£31,000-£60,000	52.9
£61,000-££90,000	17.5
> £91,000	11.9
Frequency of music listening, %	
Rarely	5.6
A couple of times a week	17.2
Daily < 1 hour	34.4
Daily 1-2 hours	29.9
Daily 3-5 hours	8.6
Daily 5+ hours	4.3
Genre of music listened to, %	
Jazz	21.0
Pop	93.7
Rock	57.7
Classical	34.2
Folk	22.8
R&B	42.8

The average wellbeing score at T1 was 24.1 (SD=3.9, range 11.25-35), at T2 was 23.9 (SD=4.3, range 7-35) and at T3 was 23.8 (SD=4.1, range 7-35) (Table 2). In order to calculate the change in wellbeing amongst these women, we analysed the difference in scores from T1 to T2 and T1 to T3. 39.2% of mothers experienced a decrease or at least 1 point in their wellbeing from T1 to T2, while 30.4% experienced no change and 30.4% experienced an improvement of at least 1 point. From T1 to T3, 41.4% of mothers experienced a decrease or at least 1 point in their wellbeing, while 25.7% experienced no change and 32.9% experienced an improvement of at least 1 point.

Table 2: Levels of wellbeing and postnatal depression during pregnancy (T1), 0-3 months post-birth (T2) and 4-6 months post-birth (T3).

	T1	T2	T3
Wellbeing			
Poor (<22)	29.4%	31.1%	31.6%
Below average (22-24)	25.6%	24.1%	25.4%
Average (25-26)	23.5%	21.3%	20.9%
Good (27-28)	10.1%	11.7%	13.7%
Excellent (29-35)	11.4%	11.9%	8.5%
Depression			
EPDS<10	74.7%	72.2%	73.3%
EPDS>=10	25.3%	27.9%	26.7%

As with wellbeing, we calculated the change in symptoms of PND from T1 to T2 and T1 to T3 (table 2). 43.6% of mothers experienced an increase in the number of symptoms of PND they were

For peer review only - http://bmjopen.bmj.com/site/about/guidelines.xhtml

For peer review only

experiencing from T1 to T2, while 11.2% experienced no change, and 45.3% of mothers experienced an improvement in symptoms. From T1 to T3, 56.4% of mothers experienced an increase in the number of symptoms of PND they were experiencing, while 11.6% experienced no change and 43.6% experienced an improvement in symptoms. In terms of the interaction between wellbeing and symptoms of PND, there was a large correlation between the two at T1 (r=-0.67, p<.001), T2 (r=-0.76, p<.001) and T3 (r=-0.77, p<.001).

Regression results: Listening to music while pregnant was associated with higher raw wellbeing scores 0-3 months post-birth, even when accounting for potential confounding variables, with greater

	Wellbeing	Symptoms of PND

frequency associated with greater effects (B=0.40, SE=0.15, 95%CI 0.10 to 0.70) (see Table 3). However, our exploratory analyses showed that effects were not evident 4-6 months post-birth. There was also an association between listening to music while pregnant and raw scores of symptoms of PND, even when accounting for potential confounding variables, with more frequent listening to music during pregnancy associated with lower symptoms of PND in the first 3 months post-birth (B=-0.39, SE=0.19, 95%CI -0.76 to -0.03). As with wellbeing, these results were no longer evident by months 4-6 post-birth.

Table 3: Associations between listening to music during pregnancy on wellbeing and symptoms of postnatal depression post-birth

Further analyses: Sensitivity analyses of the wellbeing regression models explored the potential moderating effect of mental health during pregnancy. Our analyses showed there was a significant interaction between listening and baseline wellbeing (B=-0.10 SE=0.04, 95%CI -0.17 to -0.02), with a two-way contour graph showing that listening to music particularly seemed to support those with lower wellbeing at baseline (see Supplementary Figure 1A). There was also a significant interaction between listening and baseline depression (B=-0.09, SE=0.04, 95%CI -0.17 to -0.01), with a two-way contour graph showing that listening to music particularly seemed to support those who were showing symptoms of PND (evidenced with a score of 10+ at baseline) (see Supplementary Figure 1B).

For peer review only

For peer review only - http://bmjopen.bmj.com/site/about/guidelines.xhtml

**Months 0-3 post birth (n=395)**								
	B	SE	95% CI	p	B	SE	95% CI	p
Model 1	**0.63**	**0.19**	**0.26 – 1.00**	**.001**	**-0.52**	**0.23**	**-0.98 – -0.06**	**.028**
	**R^2=0.03, F(1, 393)=11.44, p=.001**				**R^2=0.01, F(1,393)=4.74, p=.03**			
Model 2	**0.40**	**0.15**	**0.10 - 0.70**	**.01**	**-0.39**	**0.19**	**-0.76 - -0.03**	**.036**
	**R^2=0.43, F(18,376)=15.97, p<.001**				**R^2=0.39, F(18,376)=9.58, p<.001**			
**Months 3-6 post birth (n=299)**								
	B	SE	95% CI	p	B	SE	95% CI	p
Model 1	0.33	0.21	-0.079 – 0.74	.11	-0.11	0.24	-0.59 – 0.36	.63
	R^2=0.01, F(1,305)=2.17, p=.14				R^2=0.003, F(1,301)=0.86, p=.35			
Model 2	0.12	0.16	-0.20 – 0.43	.47	-0.02	0.20	-0.41 – 0.36	.90
	R^2=0.44, F(18,284)=11.22, p<.001				R^2=0.37, F(18,280)=7.19, p<.001			

*Model 1: unadjusted; Model 2: adjusted for baseline wellbeing/depression, mother's age, maternal education status, household income and number of previous children, as well as how many weeks the baby was post-birth, and the mother's marital status at T2, whether she was working at T1 or T2 and whether her partner was working at T2 and previous histories of both anxiety and depression. *p<.05, **p<.01, ***p<.001*

Finally, in order to try and ascertain whether listening to music led to changes in mental health or whether mental health led to changes in listening habits, we ran additional analyses reversing the variable order. While this does not confirm potential causal mechanisms, it can give an indication as to whether mental health can predict listening behaviours and therefore support hypotheses about temporal precedence. There was no evidence that levels of wellbeing during pregnancy were associated with the likelihood of listening to music either 3 or 6 months post-birth. However, there was some indication that depression symptoms in the final trimester of pregnancy were associated with listening habits 3 months post-birth (β=-0.03, SE=0.01, 95%CI -0.05 to -0.01, p=.003).

Discussion

This study explored associations between listening to music in the final trimester of pregnancy and mental health and wellbeing in mothers post-birth. Listening was found to be associated with higher levels of wellbeing and reduced symptoms of PND in the first 3 months post-birth, even when adjusting for baseline mental health and potential confounding variables. These results appear to be particularly found amongst women with lower levels of wellbeing at baseline. These findings echo the few existing studies in showing that listening to music is associated with better mental health in the perinatal period (30,31). However, to the authors' knowledge, this is the first study to show that listening to music during pregnancy is longitudinally associated with better mental health post-birth.

Across both symptoms of PND and wellbeing, however, associations were only found for the first 3 months post-birth, and had disappeared by the second quartile post-birth. The hypnosis study previously described also found results within the first 3 months post-partum (weeks 2 and 10) but did not measure beyond this, so there is little data available against which to benchmark these findings (16). Nevertheless, the immediate period post-birth has been highlighted as being of particular

For peer review only - http://bmjopen.bmj.com/site/about/guidelines.xhtml

challenge for new mothers, with the transition into assuming maternal tasks and adjusting to the new role lasting until around the third month postpartum (13,34). It is possible, therefore, that any effects of music listening during the prenatal period are of most value during this transition period, but become less significant once mothers and their babies become more settled.

A key question is how listening to music is associated with better mental health and wellbeing in the postnatal period. There are a number of potential explanations. First, studies involving psychological tests, neuroimaging, biomarker analyses and ethnographic observations have shown that listening to music can have marked effects on stress and anxiety (25). Specifically in relation to pregnant women, listening to music for just 30 minutes can reduce cortisol levels and anxiety (29). Wider studies involving listening to music have shown it to be particularly effective at reducing psychological and physiological responses to stress, especially when people deliberately listen to music in order to help them relax (35,36). This effect of music on stress has in turn been linked specifically through to theories around wellbeing (25,37), with a wide literature linking stress and anxiety with both mental health and wellbeing (38,39). It is proposed that high levels of anxiety might hinder women's adaptation to motherhood in the initial postpartum period, with negative effects on wellbeing (40). Consequently, it is possible that the relaxing effects of listening to music during the pregnancy period helps to act as a buffer for feelings of stress and anxiety, thereby supporting mothers in maintaining their adaptation and leading to enhanced wellbeing.

Another potential explanation relates to the effects of music on mood. Mood regulation has been identified as one of the prime reasons why people listen to music, with models of mood regulation by music highlighting its effects on mood-related subjective experience (including the intensity and clarity of moods), physiological responses (such as energy levels and movement) and behaviours (such as their ability to express emotions) (41). Music listening has been found to modulate depression and wellbeing (42,43). Early low mood during the prenatal period is directly associated with lower wellbeing and postnatal depression post-birth (44), leading to propositions that interventions that deliberately attempt to cultivate positive emotions, such as relaxation therapies and interventions focused on finding positive meaning, could directly optimise health and wellbeing in this population. Consequently, it is possible that another route by which listening to music in the third trimester of pregnancy is associated with improvements in mental health and wellbeing is via enhancing mood.

Finally, a third explanation is that listening to music in itself did not have an effect post-birth but did enhance coping skills in women while they were still pregnant, which in turn led to higher wellbeing post-birth. Music listening has been linked with both problem-oriented coping and emotion-oriented coping, specifically with results showing that problem-oriented coping by music listening in women is

For peer review only - http://bmjopen.bmj.com/site/about/guidelines.xhtml

linked to lower depression levels (45). Life transitions (such as the perinatal period) depend on both health and wellbeing and also on appraisal and coping responses. In the hypnosis study previously mentioned, the authors proposed that the intervention during pregnancy helped mothers to maintain and enhance their wellbeing whilst pregnant, which in turn influenced their appraisal of the perinatal transition period and supported their coping responses (16). It is possible that a similar process took place through listening to music, with listening to music supporting coping in the prenatal period, which encouraged mothers' own coping skills, which in turn led to better coping during and post-birth and consequent higher wellbeing. Indeed, even just in relation to the birth, a number of birth preparation courses focus on relaxation and mood optimisation, which have been shown to lead to less negative affect and better coping during labour and delivery (46). Given that a significant predictor of PND is the birth experience, enhanced coping prior to the birth, perhaps through music listening, could be an important factor in postnatal wellbeing (47).

This study has a number of limitations. First, the study followed a cohort of women rather than being interventional, so it is not possible to confirm causality. However, the study had a longitudinal design, there was no evidence of reverse causality in relation to wellbeing, there are plausible proposed mechanistic explanations, and there is a strong body of previous literature causally linking music and mental health in other populations. So this study provides promising preliminary evidence that remains to be tested in a future experimental design. A second limitation is that the population in this study was not nationally representative. Nevertheless, there was a clear spread of participants from varying socio-economic backgrounds as well as variations in the levels of exposure and outcome variables. So the dataset provides interesting and suitable preliminary data on the longitudinal associations between music listening and mental health. Third, this study explored the impact of all music listening, not specifying particular genres. Previous research has suggested that certain genres of music (or more specifically compositional aspects of music such as its valence and arousal levels) can lead to different responses, such as variations in relaxation or mood (48). However, most of these genre-specific effects have been found in tightly-controlled lab-based studies, and literature from real-world interaction with music has suggested that musical preference might be more important in determining the effects of music (49). This study followed these real-world studies in recording what genres people did listen to but measuring the quantity of listening based on preference rather than genre. Future studies could explore the impact of different genres on mental health in the perinatal period.

In conclusion, this study provides the first preliminary evidence that listening to music during the third trimester of pregnancy could be protective against symptoms of PND and low wellbeing in the first three months post-birth. Music listening is an attractive intervention in that it is readily available to people from all echelons of society regardless of socioeconomic status, educational attainment or

For peer review only - http://bmjopen.bmj.com/site/about/guidelines.xhtml

cultural background. It can be carried out in a range of contexts so is not restricted to particular places or times. It is also inexpensive: indeed the majority of women in the Western countries have access to recorded music already. Finally, there are no obvious side effects from listening to music. Consequently, listening to music could be recommended as a way of supporting pregnant women; in particular those who demonstrate low wellbeing or symptoms of PND.

Authors' contributions

DF and RP designed the study and collected data. DF ran the analyses and drafted the paper. Both authors critically revised the manuscript and approved it for submission.

Data sharing statement

The data in this study are available from the authors upon request.

References

1. Khalifeh H, Brauer R, Toulmin H, Howard LM. Perinatal mental health: What every neonatologist should know. Early Hum Dev. 2015 Nov 1;91(11):649–53.
2. Morrell C, Warner R, Slade P, Dixon S, Walters S, Paley G, et al. Psychological interventions for postnatal depression: cluster randomised trial and economic evaluation. The PoNDER trial. Health Technol Assess [Internet]. 2009 Jun [cited 2016 Jul 26];13(30). Available from: http://www.journalslibrary.nihr.ac.uk/hta/volume-13/issue-30
3. Cooper PJ, Murray L. Postnatal depression. BMJ. 1998 Jun 20;316(7148):1884–6.
4. Junge C, Garthus-Niegel S, Slinning K, Polte C, Simonsen TB, Eberhard-Gran M. The Impact of Perinatal Depression on Children's Social-Emotional Development: A Longitudinal Study. Matern Child Health J. 2017 Mar 1;21(3):607–15.
5. Green JM, Kafetsios K. Positive experiences of early motherhood: Predictive variables from a longitudinal study. J Reprod Infant Psychol. 1997 May 1;15(2):141–57.
6. Keyes CL. Mental Illness and/or Mental Health? Investigating Axioms of the Complete State Model of Health. J Consult Clin Psychol. 2005;73(3):539–48.
7. Boyce PM, Johnstone SJ, Hickey AR, Morris-Yates AD, Harris MG, Strachan T. Functioning and well-being at 24 weeks postpartum of women with postnatal depression. Arch Womens Ment Health. 2000 Dec 11;3(3):91–7.
8. Jomeen J. The importance of assessing psychological status during pregnancy, childbirth and the postnatal period as a multidimensional construct: A literature review. Clin Eff Nurs. 2004 Sep;8(3–4):143–55.
9. Allan C, Carrick-Sen D, Martin CR. What is perinatal well-being? A concept analysis and review of the literature: J Reprod Infant Psychol [Internet]. 2013 [cited 2017 Jun 8];31(4). Available from: http://www.tandfonline.com/doi/abs/10.1080/02646838.2013.791920
10. Beck CT. Predictors of postpartum depression: an update. Nurs Res. 2001 Oct;50(5):275–85.
11. Milgrom J, Gemmill AW, Bilszta JL, Hayes B, Barnett B, Brooks J, et al. Antenatal risk factors for postnatal depression: a large prospective study. J Affect Disord. 2008 May;108(1–2):147–57.
12. Robertson E, Grace S, Wallington T, Stewart DE. Antenatal risk factors for postpartum depression: a synthesis of recent literature. Gen Hosp Psychiatry. 2004 Jul;26(4):289–95.

For peer review only - http://bmjopen.bmj.com/site/about/guidelines.xhtml

13. Leifer M. Psychological effects of motherhood : a study of first pregnancy / Myra Leifer. New York: Praeger; 1980.
14. Elliott SA, Leverton TJ, Sanjack M, Turner H, Cowmeadow P, Hopkins J, et al. Promoting mental health after childbirth: a controlled trial of primary prevention of postnatal depression. Br J Clin Psychol. 2000 Sep;39 (Pt 3):223–41.
15. Spinelli MG. Prevention of postpartum mood disorders. In: Postpartum mood disorders. Arlington, VA, US: American Psychiatric Association; 1999. p. 217–35.
16. Guse T, Wissing M, Hartman W. The effect of a prenatal hypnotherapeutic programme on postnatal maternal psychological well□being. J Reprod Infant Psychol. 2006 May;24(2):163–77.
17. Chan MF, Wong ZY, Thayala NV. The effectiveness of music listening in reducing depressive symptoms in adults: A systematic review. Complement Ther Med. 2011 Dec 1;19(6):332–48.
18. Cassidy JW, Standley JM. The Effect of Music Listening on Physiological Responses of Premature Infants in the NICU. J Music Ther. 1995 Dec 21;32(4):208–27.
19. Pelletier CL. The effect of music on decreasing arousal due to stress: a meta-analysis. J Music Ther. 2004 Fall;41(3):192–214.
20. Siedliecki SL, Good M. Effect of music on power, pain, depression and disability. J Adv Nurs. 2006 Jun 1;54(5):553–62.
21. Hsu W-C, Lai H-L. Effects of music on major depression in psychiatric inpatients. Arch Psychiatr Nurs. 2004 Oct 1;18(5):193–9.
22. Chan MF, Chan EA, Mok E, Kwan Tse FY. Effect of music on depression levels and physiological responses in community-based older adults. Int J Ment Health Nurs. 2009 Aug 1;18(4):285–94.
23. Laukka P. Uses of music and psychological well-being among the elderly. J Happiness Stud. 2007 Jun 1;8(2):215.
24. Haake AB. Individual music listening in workplace settings An exploratory survey of offices in the UK. Music Sci. 2011 Mar 1;15(1):107–29.
25. Vastfjall D, Juslin PN, Hartig T. Music, subjective wellbeing, and health: the role of everyday emotions. In: MacDonald R, Kreutz G, Mitchell L, editors. Music, Health, and Wellbeing. Oxford University Press; 2013.
26. McCaffrey R. Music Listening: Its Effects in Creating a Healing Environment. J Psychosoc Nurs Ment Health Serv. 2008 Oct 1;46(10):39–44.
27. Batt-Rawden KB, DeNora T, Ruud E. Music Listening and Empowerment in Health Promotion: A Study of the Role and Significance of Music in Everyday Life of the Long-term Ill. Nord J Music Ther. 2005 Jul 1;14(2):120–36.
28. Skånland MS. Everyday music listening and affect regulation: The role of MP3 players. Int J Qual Stud Health Well-Being [Internet]. 2013 [cited 2017 Jun 14];8. Available from: /pmcc/articles/PMC3740499/?report=abstract
29. Ventura T, Gomes MC, Carreira T. Cortisol and anxiety response to a relaxing intervention on pregnant women awaiting amniocentesis. Psychoneuroendocrinology. 2012 Jan;37(1):148–56.
30. Nwebube C, Glover V, Stewart L. Prenatal listening to songs composed for pregnancy and symptoms of anxiety and depression: a pilot study. BMC Complement Altern Med. 2017 May 8;17(1):256.
31. Fancourt D, Perkins R. Associations between singing to babies and symptoms of postnatal depression, wellbeing, self-esteem and mother-infant bond. Public Health. 2017 Apr 1;145:149–52.
32. Cox JL, Holden JM, Sagovsky R. Detection of postnatal depression. Development of the 10-item Edinburgh Postnatal Depression Scale. Br J Psychiatry. 1987 Jun 1;150(6):782–6.
33. Stewart-Brown S, Tennant A, Tennant R, Platt S, Parkinson J, Weich S. Internal construct validity of the Warwick-Edinburgh Mental Well-being Scale (WEMWBS): a Rasch analysis using data from the Scottish Health Education Population Survey. Health Qual Life Outcomes. 2009;7:15.

For peer review only - http://bmjopen.bmj.com/site/about/guidelines.xhtml

34. Pridham KF, Chang AS. Transition to being the mother of a new infant in the first 3 months: maternal problem solving and self-appraisals. J Adv Nurs. 1992 Feb;17(2):204–16.
35. Krout RE. The effects of single-session music therapy interventions on the observed and self-reported levels of pain control, physical comfort, and relaxation of hospice patients. Am J Hosp Palliat Med. 2001 Dec;18(6):383–90.
36. Linnemann A, Strahler J, Nater UM. The stress-reducing effect of music listening varies depending on the social context. Psychoneuroendocrinology. 2016 Oct 1;72:97–105.
37. Krout RE. Music listening to facilitate relaxation and promote wellness: Integrated aspects of our neurophysiological responses to music. Arts Psychother. 2007;34(2):134–41.
38. Iwata M, Ota KT, Duman RS. The inflammasome: pathways linking psychological stress, depression, and systemic illnesses. Brain Behav Immun. 2013 Jul;31:105–14.
39. Vedhara K, Miles J, Bennett P, Plummer S, Tallon D, Brooks E, et al. An investigation into the relationship between salivary cortisol, stress, anxiety and depression. Biol Psychol. 2003 Feb;62(2):89–96.
40. Pond E, Kemp V. A comparison between adolescent and adult women on prenatal anxiety and self-confidence. Matern Child Nurs J. 1992;20(1):11–20.
41. Saarikallio S, Erkkilä J. The role of music in adolescents' mood regulation. Psychol Music. 2007 Jan 1;35(1):88–109.
42. Chin T, Rickard NS. Emotion regulation strategy mediates both positive and negative relationships between music uses and well-being. Psychol Music. 2014 Sep 1;42(5):692–713.
43. Lai Y-M. Effects of Music Listening on Depressed Women in Taiwan. Issues Ment Health Nurs. 1999 Jan 1;20(3):229–46.
44. Fleming A, N. Ruble D, L. Flett G, L. Shaul D. Postpartum Adjustment in First-Time Mothers: Relations Between Mood, Maternal Attitudes, and Mother-Infant Interactions. Dev Psychol. 1988 Jan 1;24:71–81.
45. Miranda D, Claes M. Music listening, coping, peer affiliation and depression in adolescence. Psychol Music. 2009 Apr 1;37(2):215–33.
46. Duncan S, J. Markman H. Intervention programs for the transition to parenthood: Current status from a prevention perspective. 2017 Oct 26;
47. Green JM. Expectations and experiences of pain in labor: findings from a large prospective study. Birth Berkeley Calif. 1993 Jun;20(2):65–72.
48. Labbe E, Schmidt N, Babin J, Pharr M. Coping with stress: the effectiveness of different types of music. Appl Psychophysiol Biofeedback. 2007 Dec;32(3–4):163–8.
49. Fancourt D, Ockelford A, Belai A. The psychoneuroimmunological effects of music: A systematic review and a new model. Brain Behav Immun. 2014 Feb;36:15–26.

For peer review only

FOOTNOTE TO SUPPLEMENTARY FIGURE: These contour plots show the relationship between baseline mental health and frequency of music listening and mental health post-birth. Darker regions indicate higher wellbeing and higher depression scores post-birth. For wellbeing, these higher response values seem to form a ridge running from the upper left to the lower right of the graph, suggesting that more frequent music listening is associated with higher wellbeing post-birth even amongst those with lower wellbeing during pregnancy. For depression, higher response values seem to form a ridge

For peer review only - http://bmjopen.bmj.com/site/about/guidelines.xhtml

running from the lower left to the upper right of the graph, suggesting that more frequent music listening is associated with lower depression post-birth even amongst those with higher depression during pregnancy.

For peer review only

For peer review only - http://bmjopen.bmj.com/site/about/guidelines.xhtml

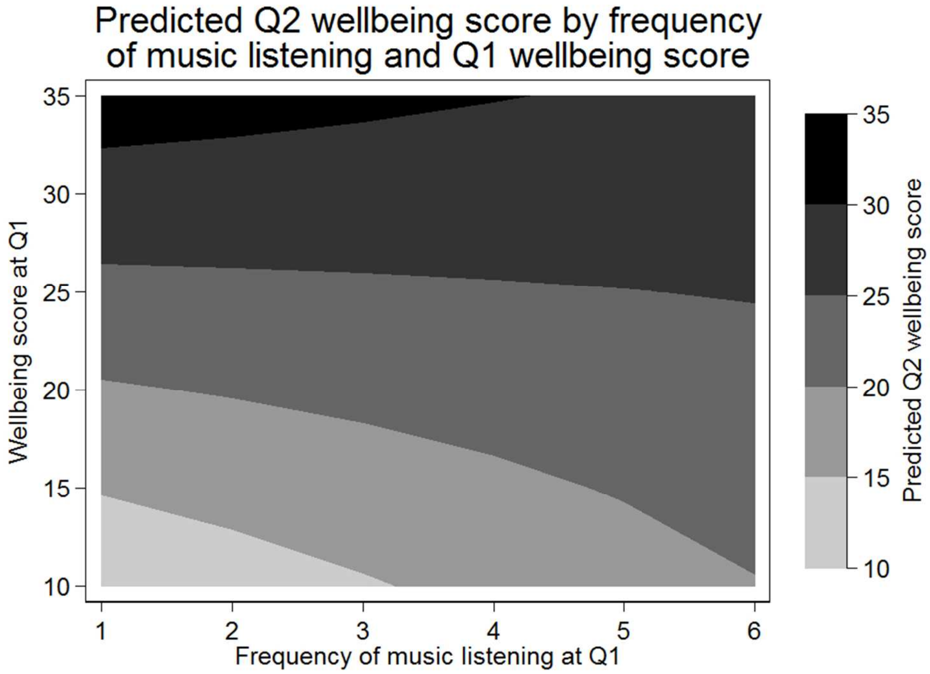

Supplementary Figure 1A

These contour plots show the relationship between baseline mental health and frequency of music listening and mental health post-birth. Darker regions indicate higher wellbeing and higher depression scores post-birth. For wellbeing, these higher response values seem to form a ridge running from the upper left to the lower right of the graph, suggesting that more frequent music listening is associated with higher wellbeing post-birth even amongst those with lower wellbeing during pregnancy. For depression, higher response values seem to form a ridge running from the lower left to the upper right of the graph, suggesting that more frequent music listening is associated with lower depression post-birth even amongst those with higher depression during pregnancy.

356x258mm (72 x 72 DPI)

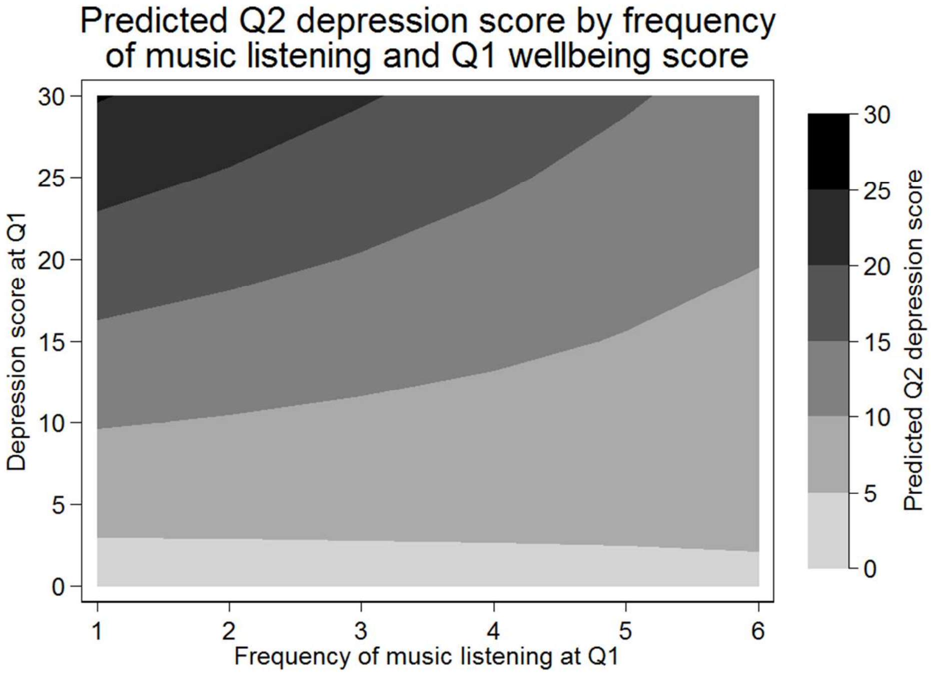

Supplementary Figure 1B

356x258mm (72 x 72 DPI)

For peer review only

STROBE Statement—Checklist of items that should be included in reports of ***cohort studies***

	Item No	Recommendation	Page
**Title and abstract**	1	(*a*) Indicate the study's design with a commonly used term in the title or the abstract	1
		(*b*) Provide in the abstract an informative and balanced summary of what was done and what was found	2
**Introduction**			
Background/rationale	2	Explain the scientific background and rationale for the investigation being reported	4-5
Objectives	3	State specific objectives, including any prespecified hypotheses	5
**Methods**			
Study design	4	Present key elements of study design early in the paper	6
Setting	5	Describe the setting, locations, and relevant dates, including periods of recruitment, exposure, follow-up, and data collection	6
Participants	6	(*a*) Give the eligibility criteria, and the sources and methods of selection of participants. Describe methods of follow-up	6
		(*b*) For matched studies, give matching criteria and number of exposed and unexposed	
Variables	7	Clearly define all outcomes, exposures, predictors, potential confounders, and effect modifiers. Give diagnostic criteria, if applicable	6-7
Data sources/ measurement	8*	For each variable of interest, give sources of data and details of methods of assessment (measurement). Describe comparability of assessment methods if there is more than one group	6-7
Bias	9	Describe any efforts to address potential sources of bias	7-8
Study size	10	Explain how the study size was arrived at	
Quantitative variables	11	Explain how quantitative variables were handled in the analyses. If applicable, describe which groupings were chosen and why	6-7
Statistical methods	12	(*a*) Describe all statistical methods, including those used to control for confounding	7
		(*b*) Describe any methods used to examine subgroups and interactions	7-8
		(*c*) Explain how missing data were addressed	8
		(*d*) If applicable, explain how loss to follow-up was addressed	8
		(*e*) Describe any sensitivity analyses	7-8
**Results**			
Participants	13*	(a) Report numbers of individuals at each stage of study—eg numbers potentially eligible, examined for eligibility, confirmed eligible, included in the study, completing follow-up, and analysed	6
		(b) Give reasons for non-participation at each stage	
		(c) Consider use of a flow diagram	
Descriptive data	14*	(a) Give characteristics of study participants (eg demographic, clinical, social) and information on exposures and potential confounders	6
		(b) Indicate number of participants with missing data for each variable of interest	N/A
		(c) Summarise follow-up time (eg, average and total amount)	6
Outcome data	15*	Report numbers of outcome events or summary measures over time	8-9
Main results	16	(*a*) Give unadjusted estimates and, if applicable, confounder-adjusted estimates and their precision (eg, 95% confidence interval). Make clear which	8-9

For peer review only

		confounders were adjusted for and why they were included	
		(*b*) Report category boundaries when continuous variables were categorized	
		(*c*) If relevant, consider translating estimates of relative risk into absolute risk for a meaningful time period	
Other analyses	17	Report other analyses done—eg analyses of subgroups and interactions, and sensitivity analyses	9-10
**Discussion**			
Key results	18	Summarise key results with reference to study objectives	10
Limitations	19	Discuss limitations of the study, taking into account sources of potential bias or imprecision. Discuss both direction and magnitude of any potential bias	12
Interpretation	20	Give a cautious overall interpretation of results considering objectives, limitations, multiplicity of analyses, results from similar studies, and other relevant evidence	12
Generalisability	21	Discuss the generalisability (external validity) of the study results	12
**Other information**			
Funding	22	Give the source of funding and the role of the funders for the present study and, if applicable, for the original study on which the present article is based	3

*Give information separately for exposed and unexposed groups.

Note: An Explanation and Elaboration article discusses each checklist item and gives methodological background and published examples of transparent reporting. The STROBE checklist is best used in conjunction with this article (freely available on the Web sites of PLoS Medicine at http://www.plosmedicine.org/, Annals of Internal Medicine at http://www.annals.org/, and Epidemiology at http://www.epidem.com/). Information on the STROBE Initiative is available at http://www.strobe-statement.org.

For peer review only

For peer review only - http://bmjopen.bmj.com/site/about/guidelines.xhtml
